# K48 and K63 linkage-competed ubiquitination of BECN1 promotes circPDE4D-mediated autophagy in chronic obstructive pulmonary disease

**DOI:** 10.1038/s41419-026-08582-8

**Published:** 2026-03-19

**Authors:** Ting-Ting Chen, Ming-Yu Wang, Jia-Ying Kang, Guo-Chun Ou, Ru Wang, Da-Wei Zhang, Jing-Jing Ye, Guang-He Fei

**Affiliations:** 1 Department of Respiratory and Critical Care Medicine, First Affiliated Hospital of Anhui Medical University, Hefei, Anhui Province China; 2 Key Laboratory of Respiratory Diseases Research and Medical Transformation of Anhui Province, Hefei, Anhui Province China

**Keywords:** Macroautophagy, Non-coding RNAs, Ubiquitylation, Respiratory tract diseases

## Abstract

Chronic obstructive pulmonary disease (COPD) is a progressive inflammatory lung disease with limited clinical therapeutic effects to suspend its progression. Circular RNAs (circRNAs) possess regulatory effects in various diseases. However, circRNA-involved regulatory mechanisms in COPD are largely unknown. This study reveals the mechanism of SMURF1-mediated BECN1 ubiquitination, which is competed by Ub-K48 and Ub-K63, driving the circPDE4D-regulated autophagy. Here, circPDE4D is first identified as a downregulated circRNA in COPD. Among patients with COPD, the lower expression of circPDE4D is associated with the reduced lung function values of FEV_1_/FVC%, FEV_1_%, and MMEF_75/25_% predicted. Moreover, circPDE4D promotes autophagy and SG formation, as well as relieves inflammation in vitro and in vivo. Mechanistically, circPDE4D binds with miR545-3p to regulate SMURF1, which functions in apoptosis, autophagy, SG formation, and inflammation. Importantly, SMURF1 interacts with BECN1 to form a complex and recruits Ub-K63 to enhance K63-linked ubiquitination of BECN1, whereas it antagonizes Ub-K48 to govern BECN1 stability. In particular, circPDE4D is indispensable for the SMURF1-induced BECN1 ubiquitination and can enhance the stability of BECN1. Together, this circPDE4D-miR545-3p-SMURF1-BECN1 regulatory feedback loop underlies the circPDE4D-mediated functions and provides valuable insights into the therapeutic application potential of COPD drugs and biomarkers developed based on circPDE4D.

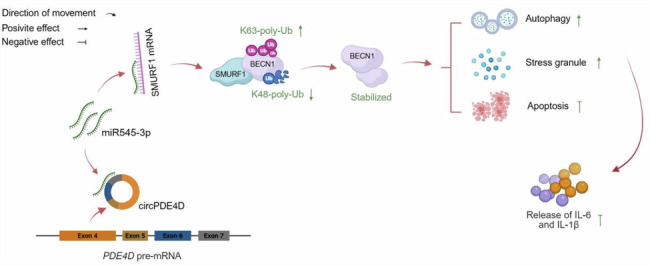

## Introduction

Chronic obstructive pulmonary disease (COPD) is a classical respiratory disease mainly caused by noxious particles and gases, especially cigarette smoke (CS), and characterized by chronic airway inflammation, persistent airflow limitation, and pathological changes involving the bronchi and alveoli [1, 2]. It remains the leading cause of morbidity, mortality, and health-care use worldwide [3]. Although there are many approaches for treating COPD, the current therapeutic effect is still poor, as it has proved difficult to inhibit the underlying inflammation [4, 5]. Therefore, clarifying the cellular and molecular mechanisms and finding new therapeutic targets for COPD is urgently needed.

Macroautophagy/autophagy is the process through which parts of the impaired cell are degraded and is vital in maintaining cell homeostasis and responding to stress [6, 7]. BECN1 (beclin 1) manages the initiation and completion of autophagy and also links to apoptosis, resulting in a crosstalk mechanism between autophagy and apoptosis [8, 9]. Stress granule (SG) is a conserved cellular strategy to promote cell survival by modulating the stress response, where SG assembly factor 1 (G3BP1) is abundant in the SGs [10–12]. Notably, researchers have demonstrated that autophagy and SG serve as quality-control pathways contributing to the inflammation regulation in various diseases, including COPD [13–17]. However, the molecular mechanism of COPD is still incompletely understood.

Ubiquitination, a critical post-translational modification, modulates diverse cellular functions by controlling protein stability and molecular interactions [18, 19]. It is well-characterized that different ubiquitin chains execute distinct cellular functions [20, 21]. The K48-linked ubiquitin chain acts as the degradation signal to trigger proteasome-mediated proteolysis, whereas the K63-linked ubiquitination regulates cellular functions by serving as a nondegradative signal [22, 23]. Interestingly, the ubiquitination of K63-linked and K48-linked may exist as a competitive event to influence protein stability and function [19, 24]. Studies reported that SMAD-specific E3 ubiquitin protein ligase 1 (SMURF1) recruits and recognizes substrates to mediate protein ubiquitination [25, 26]. However, the function of SMURF1 and the role of its related ubiquitination modification in COPD remain completely unclear.

Circular RNAs (circRNAs) are generated through a unique back-splicing mechanism involving their parental genes and possess the distinctive ring structure, which has gained significant attention in various diseases [27–29]. The powerful functions of circRNA lead to its vital role in diseases, as circRNA can serve as microRNA (miRNA) and protein sponge or decoy, act as protein scaffold, and generate unique peptides [27]. A recent study reported that circ-0000953 sponges with miR665-3p to affect podocyte injury and autophagy in diabetic nephropathy [30]. Another study showed that circCDYL promotes breast cancer progression by acting as a miR-1275 sponge [31]. Whether circRNA is involved in the regulatory mechanism of inflammation in COPD has not been fully explored.

In this study, we discovered that circPDE4D was downregulated in COPD. Its expression was associated with the lung function indicators of the FEV_1_/FVC%, FEV_1_%, and MMEF_75/25_% predicted in patients with COPD. In addition, circPDE4D suppressed cell apoptosis and inflammation, as well as promoted autophagy and SG formation in vitro and in vivo. Mechanistically, circPDE4D acted as a miR545-3p sponge to regulate SMURF1, functioning in apoptosis, autophagy, SG formation, and inflammation. Importantly, SMURF1 interacted with BECN1 to form a complex and recruited Ub-K63 to enhance K63-linked ubiquitination of BECN1, whereas it removed the K48-linked ubiquitination to stabilize BECN1 protein levels. We further found that circPDE4D increased the stability of BECN1 and was required in the process of SMURF1-regulated BECN1 ubiquitination. This ubiquitination mechanism of BECN1 protein might underlie the circPDE4D-mediated autophagy promotion. In summary, this study revealed the significant impact of BECN1 ubiquitination and circPDE4D-related functional axis on autophagy and inflammation, presenting the therapeutic potential and clinical value of circPDE4D in COPD.

## Results

### The characteristics and expression of circPDE4D in COPD

**Fig. 1:**
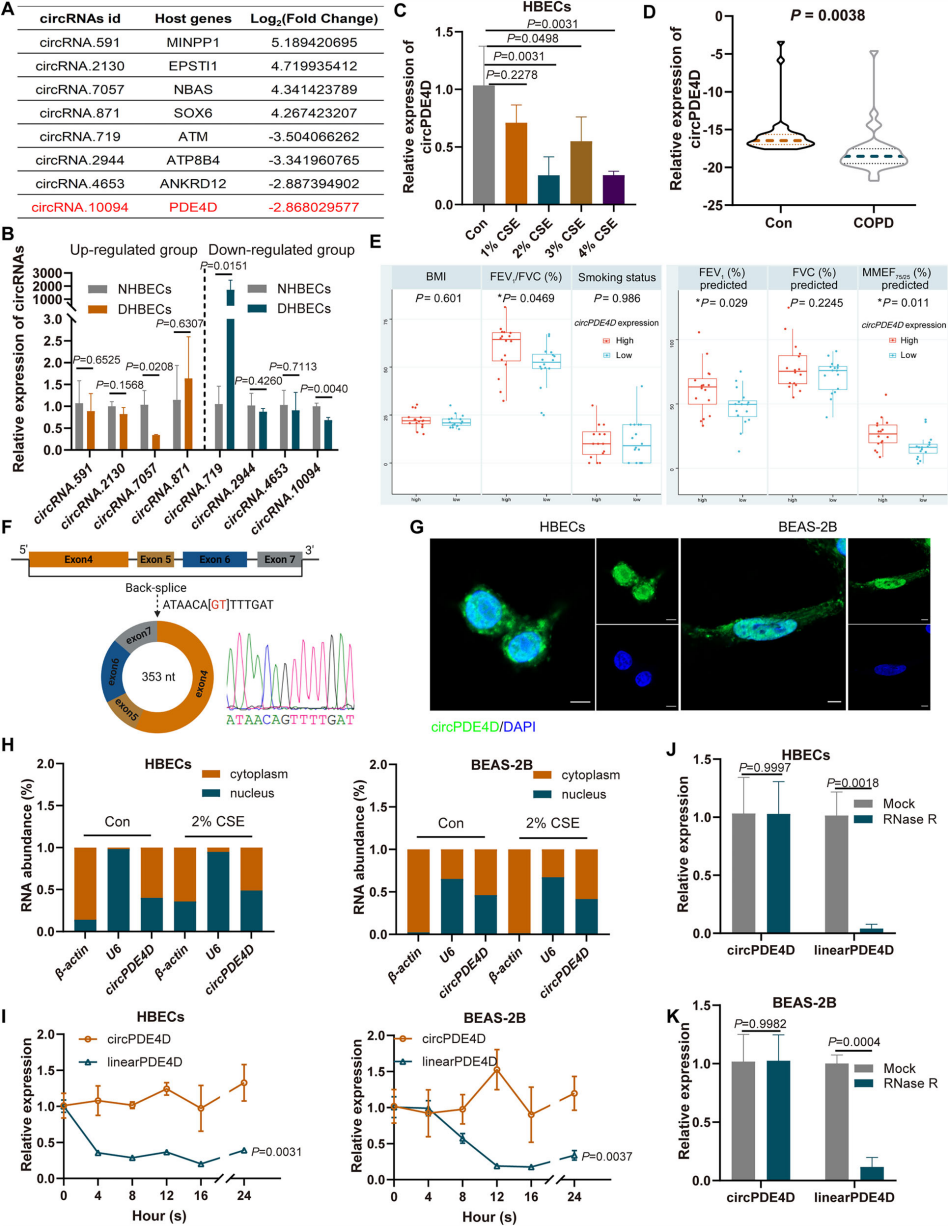
The characteristics and expression of circPDE4D in COPD. **A** Information on the top eight dysregulated circRNAs. **B** The expression of these eight circRNAs in DHBECs and NHBECs was quantified using RT-qPCR. **C** The expression levels of circPDE4D in HBEC cells with or without CSE-treatment. **D** RT-qPCR analysis revealed differential expression of circPDE4D between COPD blood samples (*n* = 32) and controls (*n* = 36). **E** Association analysis of circPDE4D expression in COPD patients with clinical information. **F** Schematic of back splicing of circPDE4D. **G** FISH with FAM-conjugated probe was employed to map circPDE4D localization. Nuclei were stained with DAPI. 5 µm scale bar. **H** RT-qPCR detection of circPDE4D expression in HBECs and BEAS-2B cells with or without CSE-treatment. **I** circPDE4D and linearPDE4D expressions were detected in cells after actinomycin D treatment. **J**, **K** The expression levels of circPDE4D and linearPDE4D were detected after RNase R treatment. Data are shown as means ± SD.

Our previous circRNA-sequencing identified differentially expressed circRNAs in peripheral blood from COPD patients; the top eight dysregulated circRNAs based on their fold-change were selected for further analysis (Fig. 1A) [32]. We next detected the expression of these circRNAs in the primary bronchial epithelial cells from patients with COPD (DHBECs) and the normal primary bronchial epithelial cells using RT-qPCR. The results showed that among these circRNAs, only circPDE4D (hsa_circ_0072568) presented consistent changes, which were consistent with the circRNA-sequencing data and showed downregulation in DHBECs cell lines (Fig. 1B). We next detected the circPDE4D levels in cigarette smoke extract (CSE) treated HBECs cells. The results showed that the expression of circPDE4D was downregulated in cells after CSE-treatment (Fig. 1C). We further examined the lower circPDE4D levels in the blood samples from COPD patients (*n* = 32) compared with control patients (*n* = 36) by performing RT-qPCR (Fig. 1D). Association analysis in COPD patients demonstrated that the expression of circPDE4D correlated with lung function indicators of the FEV_1_/FVC%, FEV_1_% predicted, and the MMEF_75/25_% predicted (Fig. 1E).

The database of circBase showed that circPDE4D was formed by reverse splicing of exons 4–7 of *PDE4D* pre-mRNA. Sanger sequencing was performed to validate the back-spliced sites of circPDE4D (Fig. 1F). The FISH localization experiment showed that circPDE4D was present in both the nucleus and cytoplasm of the indicated cells (Fig. 1G). The results of the Nuclear and Cytoplasmic separation assay were consistent with those of the FISH assay, showing that circPDE4D was distributed both in the nucleus and cytoplasm (Fig. 1H). To confirm the stability of circPDE4D, actinomycin D and RNase R assays were further structured. The results revealed that circPDE4D presented greater stability compared with linearPDE4D (Fig. 1I–K). Overall, these results suggested that circPDE4D possesses the characteristic of being highly stable in cells and is downregulated in COPD.

### CircPDE4D activates autophagy and suppresses inflammation in vitro

**Fig. 2:**
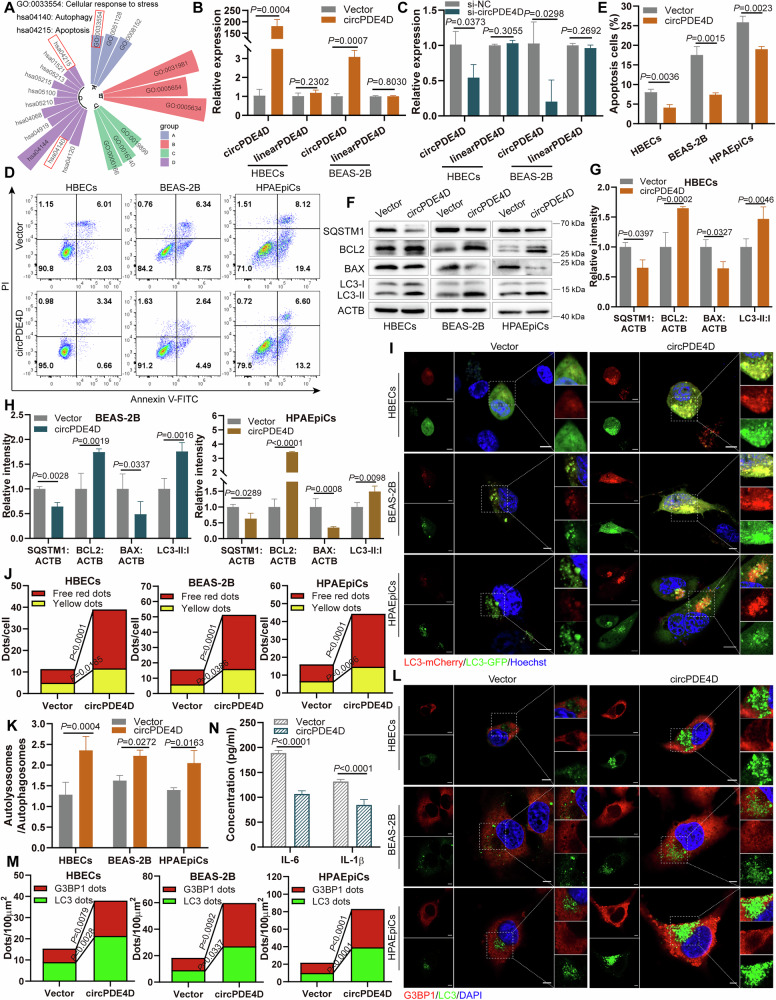
CircPDE4D activates autophagy and suppresses inflammation *in vitro.* **A** Analysis of circRNAs-related functions in COPD. **B**, **C** RT-qPCR analysis of circPDE4D and linearPDE4D expression in cells with silence and overexpression of circPDE4D. **D**, **E** Apoptosis in HBECs, BEAS-2B, and HPAEpiCs after circPDE4D overexpression was detected by flow cytometry assay and statistical quantification. **F**–**H** Western blotting with quantitative analysis of SQSTM1/p62, BCL2, BAX, and LC3 proteins in circPDE4D-overexpressing cells. **I**–**K** An abundance of LC3 dots, as reflected by red and yellow dots (merged from red and green fluorescence) in each treatment group. Nuclei were stained with Hoechst. 5 µm scale bar. **L**, **M** Immunofluorescence experiments of G3BP1 dots (red) and LC3 dots (green) in several groups. Nuclei were stained with DAPI. 5 µm scale bar. **N** The concentrations of IL-6 (in HBECs) and IL-1β (in BEAS-2B) were detected by ELISA assays in the indicated treatments. Data are shown as means ± SD.

Our previous enrichment analysis revealed that circPDE4D was correlated with apoptosis, autophagy, and cellular response to stress (Fig. 2A). To assess the functions of circPDE4D in COPD, we constructed an overexpression vector and a specific siRNA that targeted the reverse-spliced sites of circPDE4D to manipulate circPDE4D expression. After transfection of the corresponding constructs in cells, it was confirmed that circPDE4D was overexpressed and downregulated, but had no effect on the expression of linearPDE4D (Fig. 2B, C). We subsequently established CSE-induced cell models by treating cells with varying concentrations of CSE across multiple time points. The release of IL-1β and IL-6 was evaluated by ELISA assay. The results showed that after treating cells with 2%, 3%, and 4% CSE for 24 h and 32 h, the IL-1β and IL-6 levels were markedly elevated (Fig. S1A–C). In addition, we found that 3% CSE, 4% CSE, and a treatment time of 32 h resulted in a greater rate of cell death detected by the CCK-8 assay (Fig. S1D). Therefore, in subsequent studies, the condition of treating cells with 2% CSE for 24 h was adopted.

To investigate the functions of circPDE4D in apoptosis, autophagy, and stress response, we performed a series of experiments. Flow cytometry assay results revealed that circPDE4D overexpression inhibited apoptosis in HBECs, BEAS-2B, and HPAEpiCs cells (Fig. 2D, E). In contrast, circPDE4D knockdown increased cell apoptosis (Fig. S2A, B). Next, we performed a Western blot to assess the expression of key apoptosis-related proteins and key autophagy proteins. The results demonstrated that the protein levels of BCL2 and BAX were regulated by circPDE4D, with overexpression of circPDE4D promoting BCL2 expression and inhibiting BAX expression, while inhibition of circPDE4D decreased BCL2 expression and increased BAX expression in cells (Fig. 2F–H and Fig. S2C–E). During cell apoptosis, activated BAX is transferred to the mitochondrial membrane [33]. We co-localized BAX with mitochondrial membrane protein TOMM20, and the results showed that circPDE4D decreased the co-localization of BAX and TOMM20 (Fig. S2F,G). In addition, in the circPDE4D overexpression group, a reduction in the level of SQSTM1/p62 protein and an increased level of LC3 expression were observed. Conversely, SQSTM1/p62 expression was enhanced while LC3 expression was decreased in the circPDE4D knockdown group (Fig. 2F–H and Fig. S2C–E). We further evaluated autophagy flux by using the mCherry-GFP-LC3 adenovirus to infect HBECs, BEAS-2B, and HPAEpiCs cells. The results indicated that LC3 dots were activated in cells with overexpression of circPDE4D (Fig. 2I–K and Fig. S2H), while inverse patterns were detected after circPDE4D knockdown (Fig. S2H–K). Similarly, transmission electron microscopy results showed that circPDE4D promoted the formation of autolysosomes (Fig. S3A). Immunofluorescence analysis revealed increased punctate staining of G3BP1 and LC3 following circPDE4D overexpression (Fig. 2L, M), while circPDE4D silence suppressed the G3BP1 and LC3 dots (Fig. S3B, C). We next observed an obvious co-localization of circPDE4D and G3BP1 in HBECs and BEAS-2B cells (Fig. S3D). Furthermore, an ELISA assay was performed to detect the levels of inflammatory factors. The results indicated that the release of IL-1β and IL-6 was negatively regulated by circPDE4D, with reduced levels in circPDE4D-overexpressing cells (Fig. 2N) and increased expression with si-circPDE4D transfection (Fig. S3E). These results together revealed that circPDE4D activates autophagy and SG formation, and suppresses apoptosis and inflammation in vitro.

### CircPDE4D alleviates the lung structural damage and inflammation in vivo

To further investigate the biological roles of circPDE4D in COPD progression in vivo, a mouse model exposed to CS for 6 months was first established. H&E staining results observed that CS exposure induced inflammatory cell infiltration and airway wall thickening in mice lung tissues compared with controls (Fig. S4A). PAS staining results showed that the goblet cells increased in the airway tissue of CS-exposed mice, while no such cells were observed in the control group (Fig. S4A). The results of Masson staining validated that compared with air-exposed mice, CS exposure resulted in higher fibrosis in the interstitial regions and around the bronchi (Fig. S4A). In particular, the lung function detection indicated the decline of lung function in CS-exposed mice, showing with the reduced values of FEV_20_/FVC, FEV_50_/FVC, VC, MMEF, PIF, and Ti, and increased values of FRC and Te compared with those in the control group (Fig. S4B–E). Furthermore, ELISA assay of bronchoalveolar lavage fluid (BALF) in mice showed IL-6 and IL-1β levels were elevated in the CS-exposed mice than in the control mice (Fig. S4F). These observations together confirmed that CS-exposed mice develop characteristic COPD-like pathologies, including inflammation and lung structural damage.

**Fig. 3:**
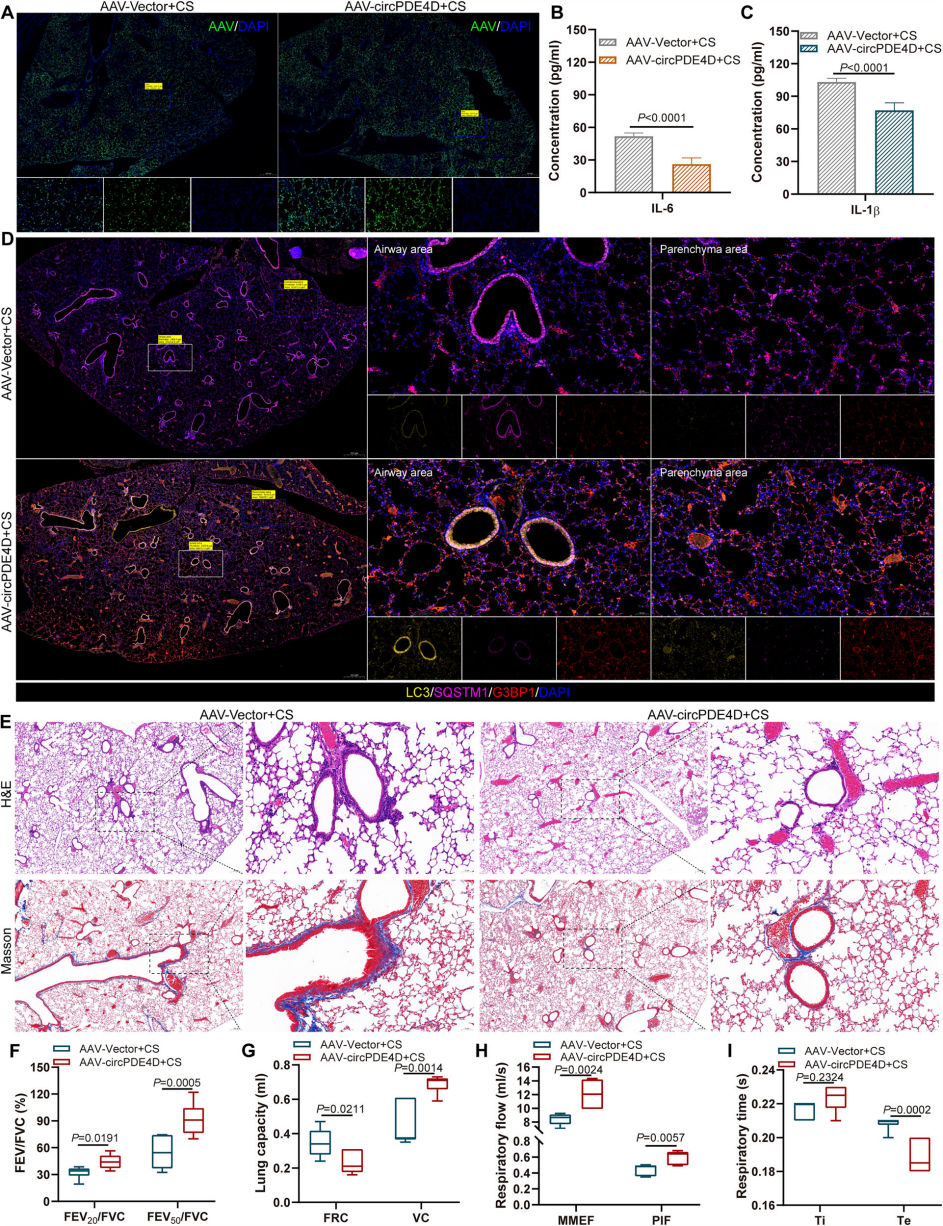
CircPDE4D alleviates the lung structural damage and inflammation *in vivo.* **A** Images showed the AAV (green) infection efficiency in mouse lungs. **B**, **C** IL-6 and IL-1β levels in AAV-Vector+CS and AAV-circPDE4D+CS groups were detected by ELISA assay (*n* = 6 mice per group). **D** Representative immunofluorescence images of LC3 (yellow), SQSTM1/p62 (cy5), and G3BP1 (red) in lung tissues from both experimental groups of mice. Nuclei were stained with DAPI. **E** Representative H&E and Masson’s staining of lung tissues from control and treated groups. **F**–**I** Lung function indices of FEV_20_/FVC, FEV_50_/FVC, FRC, VC, MMEF, PIF, Ti, and Te in the two groups. Data are shown as means ± SD.

Furthermore, we established a mouse model of circPDE4D overexpression and its control based on the model of CS exposure by employing FITC-conjugated AAV-circPDE4D and FITC-conjugated AAV-Vector, respectively. The observable green fluorescence confirmed that the AAV was successfully infected in the mouse lung (Fig. 3A). To detect the inflammation levels in mice in the two groups, we performed ELISA assays. The results validated that circPDE4D overexpression attenuated IL-1β and IL-6 release compared to vector controls (Fig. 3B, C). Overexpression of circPDE4D increased the LC3 and G3BP1 expression, and inhibited the SQSTM1/p62 expression in lung tissues of mice (Fig. 3D). In addition, compared with the AAV-Vector group, circPDE4D upregulation alleviated the destruction of lung structure in mice, manifested as reduced inflammatory cell infiltration and airway wall thickening, and decreased fibrosis levels, as observed by H&E and Masson staining (Fig. 3E). Furthermore, lung function analysis demonstrated that circPDE4D overexpression increased the values of FEV_20_/FVC, FEV_50_/FVC, VC, MMEF, PIF, and Ti, and reduced the values of FRC and Te (Fig. 3F–I). Together, these results showed that circPDE4D improves lung structural damage and inhibits inflammation, suggesting the therapeutic effect of circPDE4D in COPD.

### MiR545-3p is adsorbed by circPDE4D

**Fig. 4:**
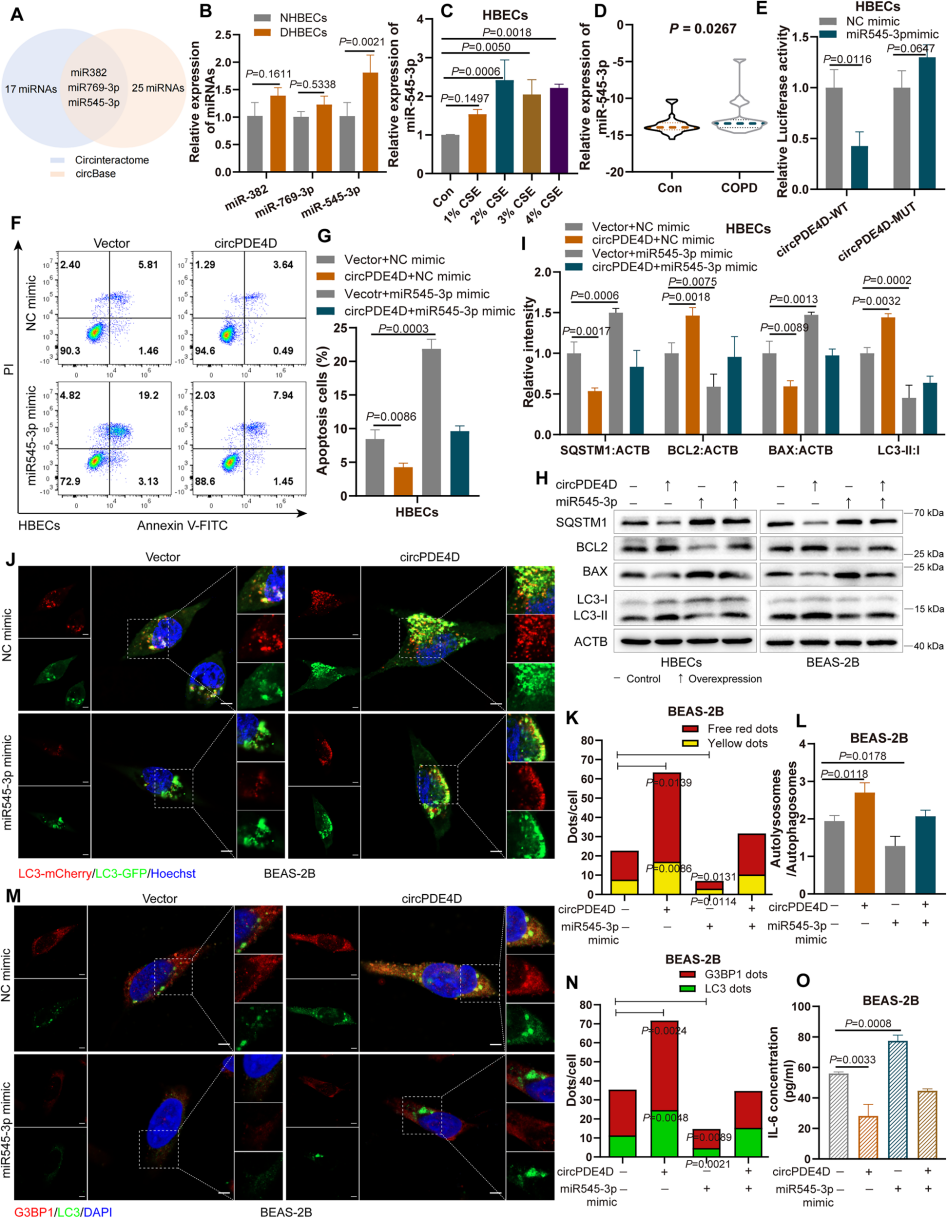
MiR545-3p is adsorbed by circPDE4D. **A** Venn diagram of the circPDE4D-targeted miRNAs. **B** Relative expressions of the three miRNAs in DHBEC cells were detected by RT-qPCR. **C** RT-qPCR detection of miR545-3p expression in CSE-treated cells. **D** The expression levels of miR545-3p in blood samples from patients of COPD (*n* = 32) and controls (*n* = 36). **E** Luciferase activities of the circPDE4D-WT and circPDE4D*-*MUT reporters transfected with miR545-3p mimic or NC mimic. **F**, **G** Flow cytometry assays and statistical quantification of apoptosis in HBEC cells with circPDE4D overexpression and co-transfected with miR545-3p mimic or controls. **H**, **I** Proteins of SQSTM1/p62, BCL2, BAX, and LC3 levels in cells in several groups based on western blotting. **J**–**L** LC3 dots as reflected by free red dots and yellow dots in cells after co-transfection with the circPDE4D plasmid with miR545-3p mimic or its control. Nuclei were stained with Hoechst. 5 µm scale bar. **M**, **N** Immunofluorescence experiments of G3BP1 dots and LC3 dots in BEAS-2B. Nuclei were stained with DAPI. 5 µm scale bar. **O** ELISA detection of IL-6 levels in BEAS-2B cells in the indicated groups. Data are shown as means ± SD.

CircRNAs, as miRNA sponges, can adsorb miRNAs to execute their functions in various diseases [27]. To explore the molecular mechanism of circPDE4D-mediated apoptosis, autophagy, SG assembly, and inflammatory factors regulation, we screened potential targeted miRNAs of circPDE4D using the prediction databases of circinteractome and circBase. The intersection analysis showed three miRNAs were identified, including miR382, miR769-3p, and miR545-3p (Fig. 4A). We then detected the expression of these three miRNAs in DHBECs. Among them, only miR545-3p showed a dysregulated change manifested as upregulation in DHBECs cells compared with NHBECs (Fig. 4B). Similar findings were detected in CSE-treated HBECs cells, as well as in the blood samples from COPD patients, showing miR545-3p was an upregulated miRNA (Fig. 4C, D). Next, we constructed dual luciferase reporter plasmids (wild-type and mutant-type) of circPDE4D to evaluate the combining capacity with miR545-3p (Fig. S5A); the luciferase activity was reduced in the circPDE4D-WT group but not in the circPDE4D-MUT group after co-transfection with miR545-3p mimic (Fig. 4E and Fig. S5B). These results indicated that circPDE4D can act as a miR545-3p sponge to bind with miR545-3p.

To assess the effects of miR545-3p on circPDE4D-regulated functions, we also validated the overexpression and inhibition of miR545-3p in cells by using the miR545-3p mimic and inhibitor, respectively (Fig. S5C). The rescue experiment results demonstrated that overexpression of miR545-3p eliminated the inhibitory effects of circPDE4D overexpression on apoptosis, while inhibition of miR545-3p attenuated the promotion of cell apoptosis induced by circPDE4D silencing (Fig. 4F, G and Fig. S5D, E). In addition, ectopic miR545-3p expression suppressed the BCL2 and LC3 protein levels and strengthened BAX and SQSTM1/p62 levels, and partly reversed the effects of circPDE4D on these proteins (Fig. 4H, I and Fig. S5F); whereas the opposite trends were observed after knocking out miR545-3p (Fig. S5G–I). Overexpression of miR545-3p promoted the co-localization of BAX and TOMM20 (Fig. S5J, K). Consistent findings were obtained through immunofluorescence staining; the circPDE4D-mediated promotional effects on LC3 dots, G3BP1, and LC3 expression were impeded by miR545-3p overexpression (Fig. 4J–N and Fig. S5L), while miR545-3p silence reversed the inhibitory effects caused by circPDE4D knockdown (Fig. S6A–F). Furthermore, circPDE4D-regulated concentrations of both IL-1β and IL-6 were also eliminated by miR545-3p (Fig. 4O and Fig. S6G). Overall, these results suggested that circPDE4D regulates cell apoptosis, autophagy, SG formation, and the release of IL-1β and IL-6 by targeting its downstream effector miR545-3p.

### SMURF1 participates in circPDE4D-targeted miR545-3p regulation axis

**Fig. 5:**
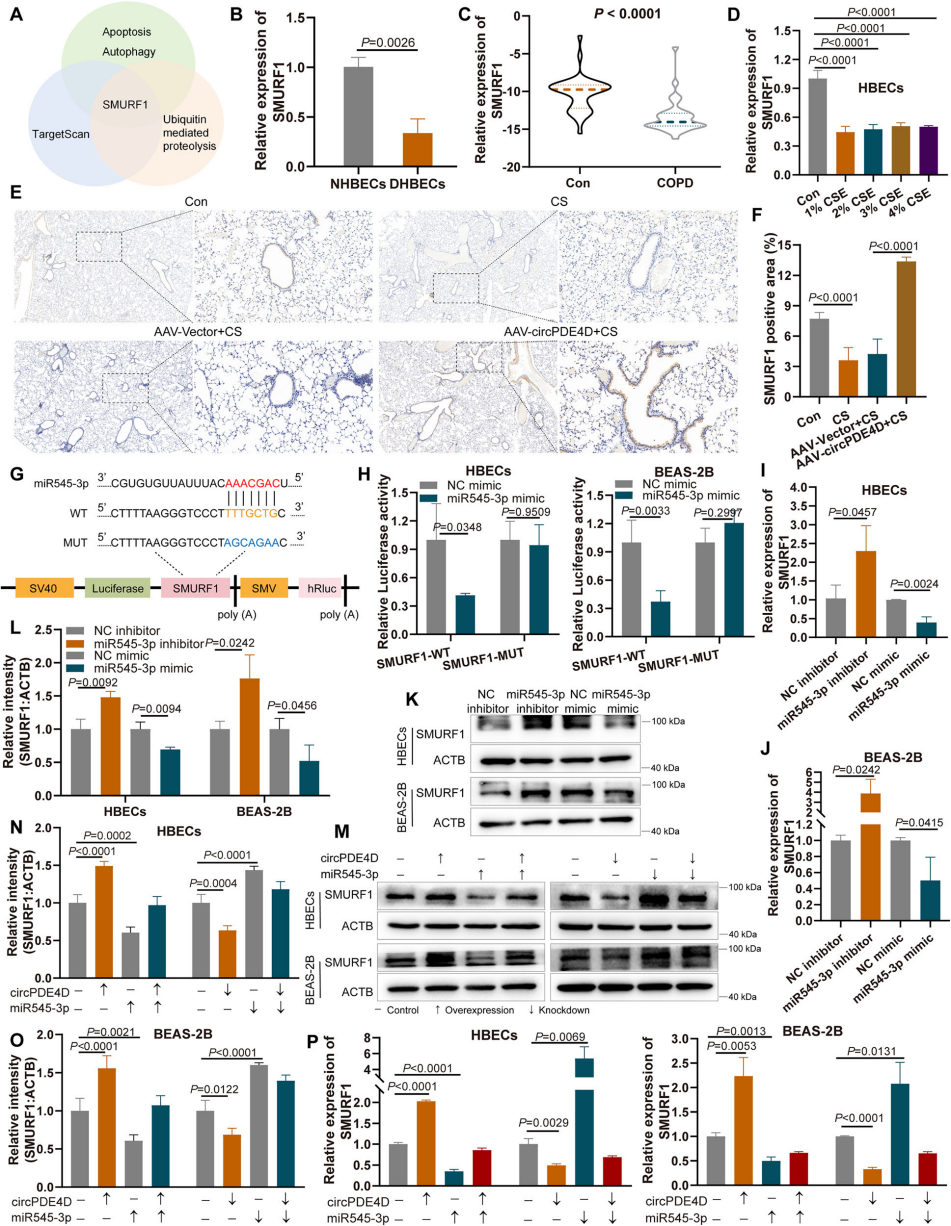
SMURF1 participates in circPDE4D-targeted miR545-3p regulation axis. **A** Venn diagram showing that *SMURF1* is a miR545-3p-targeted and function-related gene. **B** Relative expression of SMURF1 in DHBECs cells compared with NHBECs. **C** Comparative SMURF1 levels in blood between COPD (*n* = 32) and control cohorts (*n* = 36). **D** SMURF1 expression was compared between CSE-treated and control cells. **E**, **F** Representative immunohistochemical images and quantitative analysis of SMURF1 (brown) in lung sections from different treatment groups. **G** Schematic of the luciferase reporter plasmids of SMURF1-WT and MUT. **H** Relative luciferase activities of the SMURF1-WT and MUT reporters transfected with miR545-3p mimic or NC mimic. **I**–**L** Relative expressions of SMURF1 at both the mRNA and protein levels were detected by RT-qPCR and western blotting after miR545-3p overexpression and knockdown. **M**–**O** Western blotting with quantitative analysis of SMURF1 protein levels in cells transfected as indicated. **P** RT-qPCR analysis detected the expression of SMURF1 following experimental treatments. Data are shown as means ± SD.

Since miRNAs play their functions by interacting with downstream genes, we next predicted potential genes of miR545-3p-interacted using a database of TargetScan [34]. Considering the apoptosis, autophagy, and ubiquitin-mediated proteolysis (hsa04120) were circPDE4D-related functions (Fig. 2A), we performed intersection analyses of genes from TargetScan, ubiquitin-related genes, and apoptosis and autophagy-related genes. The results showed that only SMURF1 was screened (Fig. 5A). SMURF1, a member of the E3 ubiquitination ligases, serves as a critical regulator in both autophagy modulation and inflammatory response pathways [35–37]. Similar to the patterns of circPDE4D expression in COPD and CSE-treated cells, the lower expression of SMURF1 was observed in the DHBECs cells and blood samples of COPD patients, as well as in CSE-treated HBECs cells (Fig. 5B–D). We also confirmed the expression of SMURF1, which was reduced in CS-exposed mice, and positively regulated by circPDE4D (Fig. 5E, F). According to the TargetScan results, SMURF1 and circPDE4D share the same binding region of miR545-3p (Fig. 5G). The dual luciferase reporter assay showed that miR545-3p resulted in the downregulation of luciferase activity from the SMURF1-WT reporter but not the SMURF1-MUT reporter (Fig. 5H). In addition, SMURF1 was negatively regulated by miR545-3p at both the mRNA stability and protein levels (Fig. 5I–L), while these effects were reversed by circPDE4D (Fig. 5M–P). These data suggested SMURF1 interacts with miR545-3p and is indirectly regulated by circPDE4D.

### CircPDE4D-mediated functions are SMURF1-dependent

**Fig. 6:**
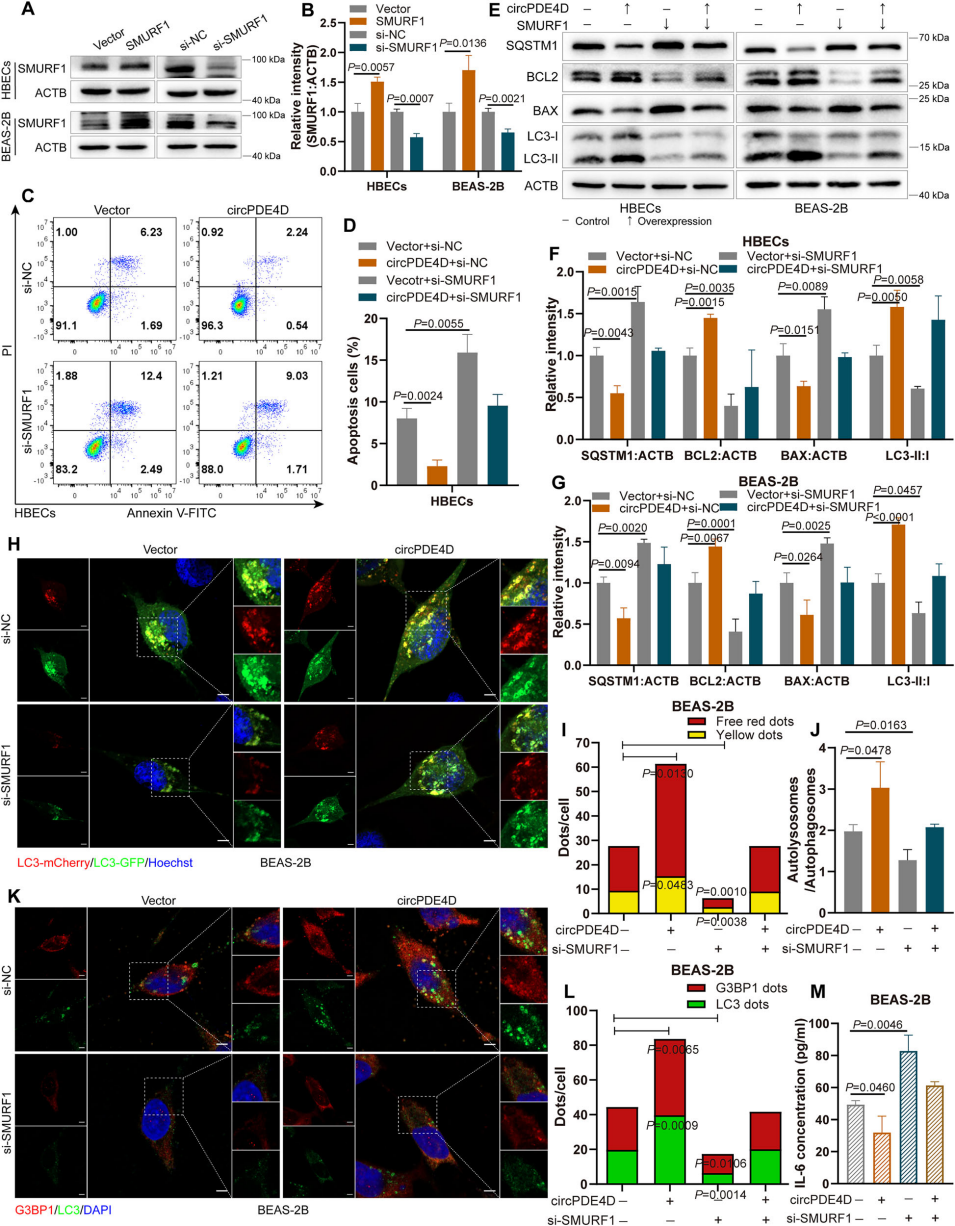
CircPDE4D-mediated functions are SMURF1-dependent. **A**, **B** Western blotting and quantitation of SMURF1 in the indicated cells after SMURF1 overexpression and knockdown. **C**, **D** Flow cytometry assay of apoptosis detection in HBECs cells with circPDE4D overexpression and co-transfected with si-SMURF1 or controls. **E**–**G** Western blotting and quantitation of SQSTM1/p62, BCL2, BAX, and LC3 protein levels in cells after designated treatments. **H**–**J** LC3 dots as reflected by free red dots and yellow dots in several groups after mCherry-GFP-LC3 transfection. Nuclei were stained with Hoechst. 5 µm scale bar. **K**, **L** Immunofluorescence experiments of G3BP1 dots and LC3 dots in BEAS-2B following the designated treatments. Nuclei were stained with DAPI. 5 µm scale bar. **M** ELISA detection of IL-6 levels in BEAS-2B cells in the indicated groups. Data are shown as means ± SD.

To further evaluate the role of SMURF1 in circPDE4D-mediated apoptosis, autophagy, SG formation, and inflammation in COPD, we modulated SMURF1 levels using an overexpression plasmid and siRNA, and confirmed the changes in SMURF1 in HBECs and BEAS-2B cells (Fig. 6A, B). Flow cytometry assay results demonstrated that SMURF1 knockdown promoted apoptosis in HBECs and BEAS-2B cells (Fig. 6C, D), while the opposite effect was observed by upregulating SMURF1 (Fig. S7A, B). In addition, the inhibition of apoptosis in cells caused by circPDE4D overexpression was rescued by silencing SMURF1 (Fig. 6C, D), while the enhancement of apoptosis with circPDE4D knockdown was abrogated by augmenting SMURF1 (Fig. S7A, B). Parallel trends were noted in western blot analysis; manipulation of SMURF1 impeded the effects of circPDE4D on BCL2 and BAX, as well as on SQSTM1/p62 and LC3 (Fig. 6E–G and Fig. S7C–E). SMURF1 knockdown promoted the co-localization of BAX and TOMM20 and inhibited the formation of autolysosomes (Fig. S7F–H). Furthermore, circPDE4D-induced LC3 dots, as well as G3BP1 and LC3 expression, were hindered with SMURF1 knockdown (Fig. 6H–L and Fig. S7I), while the reduced expression of LC3 dots, G3BP1, and LC3 in cells caused by circPDE4D silence was rescued by SMURF1 overexpression (Fig. S7J–M and Fig. S8A, B). The effects of circPDE4D on IL-1β and IL-6 expression could be reversed by SMURF1 intervention (Fig. 6M and Fig. S8C). These findings suggested that SMURF1 emerges as a pivotal modulator governing the functional output of the circPDE4D signaling pathway.

### SMURF1 positively regulates BECN1 abundance by promoting the K63-linked ubiquitination

**Fig. 7:**
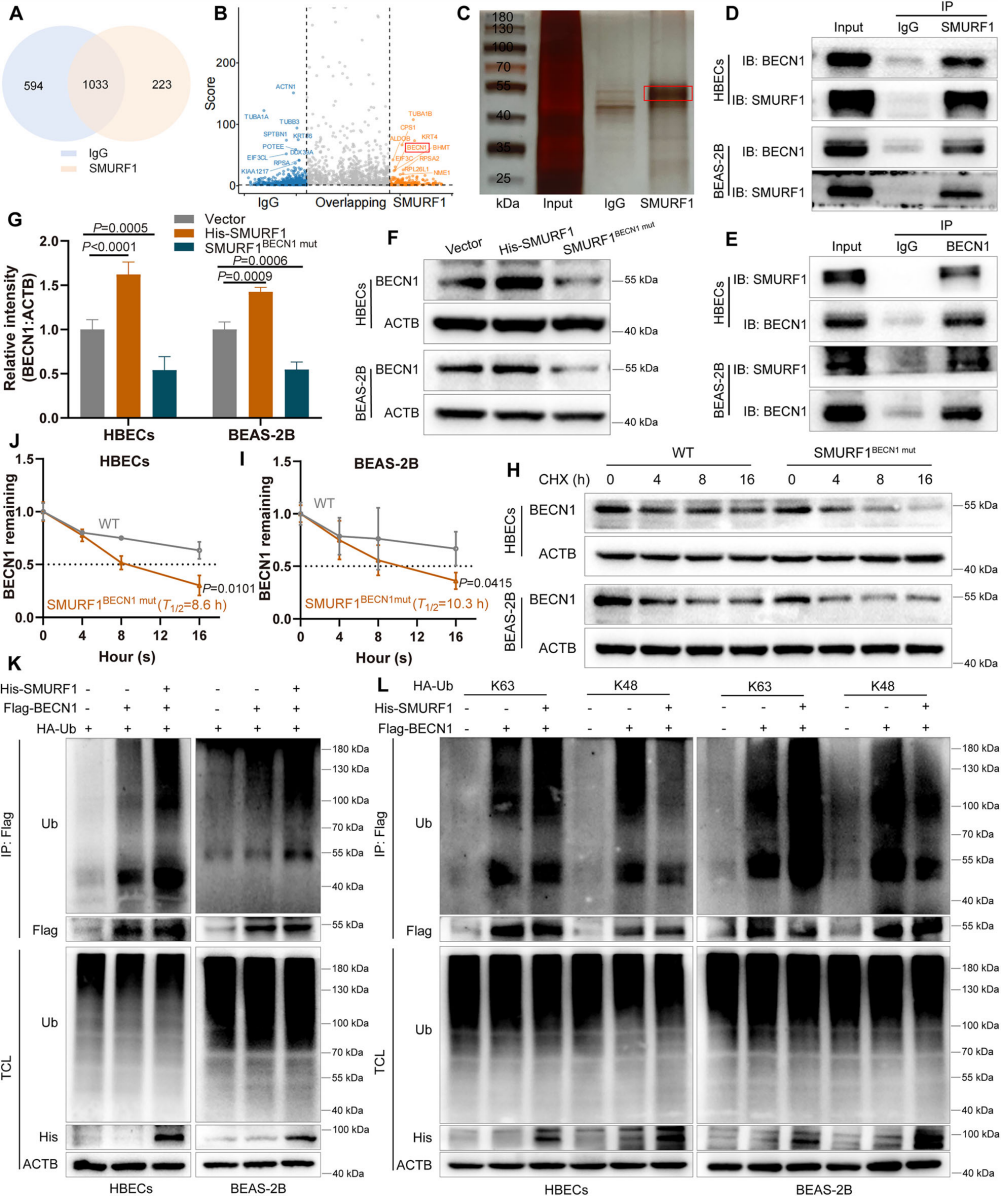
SMURF1 positively regulates BECN1 abundance by promoting the K63-linked ubiquitination. **A** Comparative protein expression visualized through a Venn diagram in IgG and anti-SMURF1 groups after detection by MS analysis. **B** Volcano plot of differentially expressed proteins of the two groups, with the top 10 candidates highlighted. **C** Silver staining experiment showing the SMURF1-interacted proteins. **D**, **E** Co-IP assay of SMURF1-BECN1 complex was conducted with specific antibodies (anti-SMURF1 or anti-BECN1) versus an IgG control. **F**, **G** Western blotting and quantitation of BECN1 in cells in the vector, His-SMURF1, and SMURF1^BECN1mut^ groups. **H**–**J** Western blotting with quantitative analysis of BECN1 protein levels at specific times in control and SMURF1^BECN1mut^ overexpression cells treated with cycloheximide (CHX) at the indicated time points. The dotted line represents the half-life of BECN1. IP and western blotting of the ubiquitination in cells with or without His-SMURF1 and Flag-BECN1 overexpression and co-transfected with HA-Ub (**K**), HA-Ub-K48 (**L**), or HA-Ub-K63 (**L**). Data are shown as means ± SD.

SMURF1, as an E3 ubiquitination ligase, regulates the stability of proteins by regulating protein ubiquitination modification [38]. To further explore the occurrence mechanism of autophagy, we performed a series of experiments. MS analysis results showed that, under the thresholds of fold-change >2 and *P*-value < 0.05, a total of 223 candidate proteins were identified in the SMURF1 group (Fig. 7A). Notably, BECN1 was identified among the top ten SMURF1-interacting proteins and is known to participate in autophagy regulation (Fig. 7B) [39]. BECN1 is a core regulatory protein in the process of autophagy and is crucial in the formation and initiation stages of autophagosomes [40]. Silver staining analysis validated the physical interaction between SMURF1 and BECN1 (Fig. 7C). Consistent results were observed in Co-IP assays (Fig. 7D, E). In addition, SMURF1 increased the BECN1 protein levels, and the SMURF1^BECN1mut^ mutant lost the effect on BECN1 expression (Fig. 7F, G). Of note, in the presence of overexpressing SMURF1^BECN1mut^ mutant, BECN1 showed reduced half-lives (Fig. 7H–J). These findings suggested that SMURF1 participates in stabilizing BECN1 by binding to BECN1, pointing to the possibility that SMURF1 antagonizes Ub-K48 to impede proteasomal degradation of BECN1.

We then examined the ubiquitination levels of BECN1, and the results showed that SMURF1 accelerated the BECN1 ubiquitination in HBECs and BEAS-2B cells (Fig. 7K). To determine the type of ubiquitin chain linkage on BECN1, we co-transfected BECN1 with two ubiquitin K-only constructs. The results demonstrated that in addition to K48-linked ubiquitination, BECN1 was also modified by K63-linked ubiquitination (Fig. 7L). Importantly, SMURF1 led to an elevated Ub-K63 in BECN1 ubiquitination in cells and an attenuated Ub-K48 (Fig. 7L). In summary, these results suggested that SMURF1 positively regulates BECN1 protein abundance by removing K48-linked ubiquitination and increasing K63-linked ubiquitination of BECN1.

### CircPDE4D is indispensable for the SMURF1-induced BECN1 ubiquitination

**Fig. 8:**
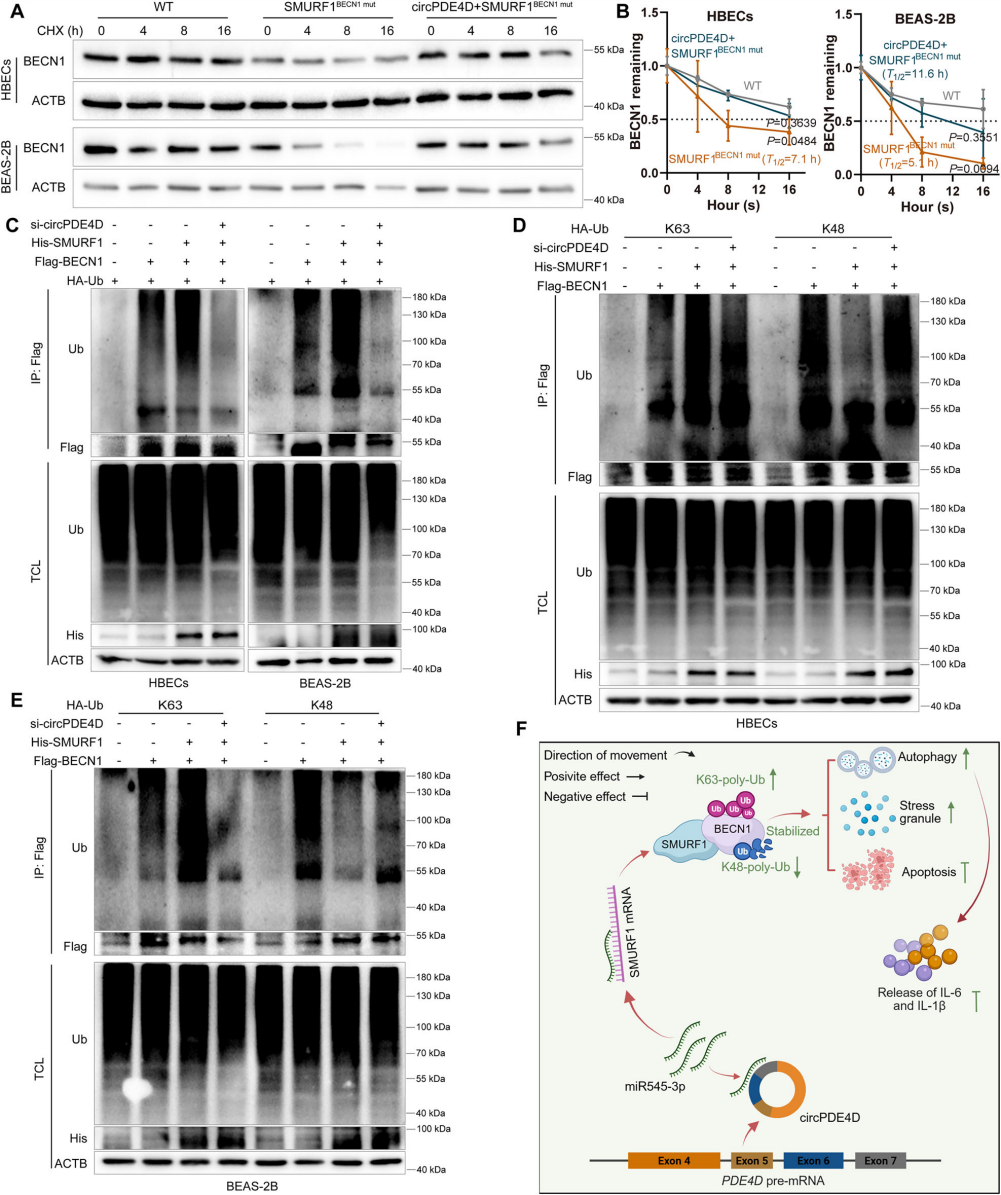
CircPDE4D is indispensable for the SMURF1-induced BECN1 ubiquitination. **A**, **B** Western blotting and quantitation of BECN1 in cells with or without SMURF1^BECN1mut^ overexpression and co-transfected with circPDE4D plasmid or its control. Cycloheximide (CHX) treats cells at the indicated time points. The dotted line represents the half-life of BECN1. **C** IP and western blotting of the ubiquitination in HA-Ub overexpressing cells transfected with or without His-SMURF1, Flag-BECN1, and si-circPDE4D. **D**, **E** Ubiquitination was detected by IP and western blotting in HA-Ub-K48 or HA-Ub-K63 overexpressing cells transfected with or without His-SMURF1, Flag-BECN1, and si-circPDE4D. Data are shown as means ± SD. **F** Schematic diagram: circPDE4D adsorbs miR545-3p to target SMURF1. SMURF1 interacts with BECN1 to recruit Ub-K63 and remove K48-linked ubiquitination of BECN1. The SMURF1-regulated competitive effect on ubiquitination of K63 linkage with K48 linkage stabilizes BECN1 protein levels. This ubiquitination mechanism is the basis of circPDE4D-mediated functions. Created by BioRender.

CircRNA participates in the ubiquitination process by influencing protein stability and the activity of E3 ubiquitination ligase [41]. Given the findings shown above, we further examined a possible role of circPDE4D in the regulation of BECN1 ubiquitination. Indeed, the reduced half-lives of BENC1 caused by the SMURF1^BECN1mut^ mutant were partly reversed by circPDE4D overexpression (Fig. 8A, B). SMURF1-induced BECN1 ubiquitination was completely impeded by silencing circPDE4D (Fig. 8C). Furthermore, the absence of circPDE4D dramatically attenuated the positive effects of K63-linked ubiquitination of BECN1 in SMURF1-overexpressing cells and abrogated the reduced K48-linked ubiquitination (Fig. 8D, E). Together, these results demonstrated that SMURF1 loses the effects on Ub-K63 and Ub-K48 regulation after circPDE4D knockdown, suggesting that circPDE4D was required for SMURF1-mediated ubiquitination of BECN1 and also regulated BECN1 stability, thereby creating a circPDE4D-miR545-3p-SMURF1-BECN1 self-reinforcing loop.

## Discussion

COPD is a progressive inflammatory lung disease, which is mostly brought on by inhaled particles, especially CS [3]. Inflammation contributes to systemic manifestations and exacerbates comorbid diseases in COPD [42]. Thus, controlling inflammation is crucial for the treatment of COPD. Ubiquitination controls protein stability and complex formation to modulate inflammatory response, and is regulated by a range of enzymes, including E3 ubiquitination ligases [43]. SMURF1 is one of the E3 ubiquitination ligases and dynamically manages the process of ubiquitination [44]. Importantly, SMURF1 also has a tight relationship with autophagy and inflammation; it has been identified as an autophagy regulator and is required for clearing damaged lysosomes and eliminating pulmonary inflammation [35–37]. These indicate that SMURF1 may influence inflammation by directing ubiquitination modification of proteins and regulating autophagy.

Although circRNA-based studies have advanced greatly, research on COPD is still lacking [45, 46]. Our study verified that circPDE4D was a stably downregulated circRNA in the biological samples of COPD patients, as well as in CSE-treated cells. Its expression level was correlated with lung function indicators of the FEV_1_/FVC% ratio, FEV_1_% predicted, and MMEF_75/25_% predicted. These findings indicate that circPDE4D may be a functional circRNA in COPD. Indeed, we further confirmed that circPDE4D-regulated apoptosis, autophagy, SG formation, and inflammation in vitro. In vivo, circPDE4D relieved lung tissue damage in mice and inhibited inflammation, indicating its potential therapeutic benefits for COPD.

Given the prominent role of circPDE4D in COPD, we next explored the circPDE4D-mediated underlying mechanism. The competitive endogenous RNA mechanism is a crucial mechanism of circRNAs for biological functions in COPD [47]. It is reported that circ0061052 can sponge miR-515-5p to be involved in the CS-induced epithelial-mesenchymal transition in COPD [48]. In our study, different databases (circinteractome and circBase) were used to screen the potential miRNAs. The initially screened miRNAs were further detected for expression levels in DHBECs, so that miR545-3p was identified for subsequent studies. Dual luciferase reporter assays demonstrated the combination of circPDE4D and miR545-3p, suggesting that circPDE4D acted as a miR545-3p sponge to execute its functions. Our study further verified circPDE4D-miR545-3p axis represents an efficient way of regulating apoptosis, autophagy, SG formation, and inflammatory factors.

MiRNA pairs with the mRNA of protein-coding genes to direct their posttranscriptional repression, thereby controlling the occurrence and development of various diseases [34]. Through bioinformatic analysis, we found *SMURF1* was the target gene of miR545-3p. Although it is well-characterized that SMURF1 is critical for ubiquitination modification, its regulatory role in COPD remains obscure. In our study, SMURF1 bound with miR545-3p and was indirectly regulated by circPDE4D. The effects of the circPDE4D-miR545-3p-SMURF1 axis on apoptosis, autophagy, SG accumulation, and inflammatory factors regulation were further verified by rescue experiments. We also found that circPDE4D-mediated biological functions were SMURF1-dependent by performing rescue experiments, suggesting that SMURF1 is a key regulator in COPD.

Our previous results showed ubiquitin-mediated proteolysis was one of the circPDE4D-related pathways. SMURF1 can recognize and bind to specific substrates to guide ubiquitination modification [49]. Thus, whether such a SMURF1-mediated ubiquitination participates in our study should be considered. BECN1, a core player in autophagy regulation, is indispensable for autophagy induction [39]. Here, BENC1 was identified by MS analysis and interacted with SMURF1 to form a SMURF1-BECN1 complex. Studies have shown that the ubiquitination of K63 linkage can compete with K48 linkage to stabilize protein levels [19, 24]. In our study, SMURF1 stabilized BECN1 protein levels by antagonizing Ub-K48 to impede proteasomal degradation of BECN1 and promoting K63-linked ubiquitination levels. These results suggest that the ubiquitination of K63 linkage may compete with that of K48 linkage on BECN1 to govern BECN1 stability. This mechanism might underlie the circPDE4D-mediated autophagy promotion. Our study further confirmed that circPDE4D was required for SMURF1-regulated BECN1 ubiquitination and also enhanced the stability of BECN1. This circPDE4D-miR545-3p-SMURF1-BECN1 regulatory feedback loop suggests that the ubiquitination process of BECN1 is undergoing dynamic regulation in cells. The SMURF1 expression levels dictate different types of ubiquitination linkage, including but not limited to K48 and K63, to impact BECN1 stability and function.

In conclusion, this study revealed that functional circPDE4D-targeted miR545-3p to regulate SMURF1, functioning in apoptosis, autophagy, SG formation, and inflammation. Interestingly, SMURF1 stabilized BECN1 protein levels by removing Ub-K48 and increasing the K63-linked ubiquitination of BECN1, which circPDE4D was necessary for this process. This circPDE4D-miR545-3p-SMURF1-BECN1 regulatory feedback loop was the basis of the circPDE4D-mediated functions in the inhibition of inflammation and prevented the deterioration of COPD. Our study reveals that circPDE4D holds great potential for therapeutic applications in inflammation, and its novel regulatory loop provides valuable insights into the progression of COPD. Targeting circPDE4D may represent a novel therapeutic strategy for COPD.

## Materials and methods

### Ethics statement

All experiments involving human blood samples were conducted according to the ethical principles and performed with the approval of the Ethics Committee of First Affiliated Hospital of Anhui Medical University (Approval No. PJ 2024-07-56). All participants provided informed consent prior to their involvement in the study.

All animal experiments were carried out with the ethical principles and approval by the Animal Ethics Committee of Anhui Medical University (Approval No. LLSC20242225).

### Human blood samples

The collection of blood samples from patients with COPD (*n* = 32) and patients in the control group (*n* = 36) was carried out following the ethical principles at the First Affiliated Hospital of Anhui Medical University. All patients who participated provided informed consent.

### CircRNA-sequencing

Briefly, we collected blood samples from COPD and non-COPD individuals and added the total RNA extraction reagent (Epizyme, YY101) into the samples immediately. The samples were then used to extract total RNA. The circRNA-sequencing analysis was performed and analyzed by the Illumina HiSeq^TM^ platform. Differential expression of circRNAs was screened with the criteria of *P*-value < 0.05 and fold-change >2.

### Cigarette smoke extract (CSE) preparation

Smoke from Marlboro cigarettes (Philip Morris, UK) was bubbled into a centrifugal tube, which contained RPMI-1640 solution (15 mL) at a stable speed through a vacuum pump. The CSE solution was filtered three times using a 0.22 µm PES membrane filter unit (Millipore, SLGPR33RB). Quality control followed the criteria that the ΔOD was between 0.9 OD and 1.2 OD under the absorbance of 320 nm and 540 nm, indicating that the solution was 100% CSE.

### Cell isolation and culture

Primary bronchial epithelial cells were isolated from COPD patients (DHBECs) and normal controls (NHBECs) as described in our previous studies [50]. Briefly, bronchial tissues were obtained by enzymatic digestion and expanded on a collagen-coated dish in BEGM medium. Finally, an immunofluorescence experiment was performed to identify the bronchial epithelial cells.

Human bronchial epithelial cells (HBECs and BEAS-2B) were obtained from the American Type Culture Collection. The HBECs cell line was grown in RPMI-1640 medium (Gibco, C11875500BT). The BEAS-2B cell line was grown in DMEM medium (Gibco, C11995500BT). Human pulmonary alveolar epithelial cells (HPAEpiCs) were obtained from OTWO Biotech and maintained in DMEM medium. Those media were supplemented with 10% FBS (Gibco, 10270–106). All cells were cultured at 37 °C in an environment with 5% carbon dioxide, free from mycoplasma contamination.

### Cell transfection

Overexpression plasmids, siRNAs, miRNA mimic/inhibitor, and the corresponding controls (GenePharma, China) underwent transfection using Nano 40^TM^ (Biomedical, B1002). The information on all constructs is listed in Table S1 (Supporting Information). Adenovirus of mCherry-GFP-LC3 (Beyotime Biotechnology, C3011) was used to treat cells at an MOI of 15 to detect autophagy flux. Images of autophagy flux were captured by a laser confocal microscope (LSM980, Carl Zeiss, Oberkochen, Germany).

### Cell treatment assays

In the CSE-treated experiment, 100% CSE was diluted to several concentrations of 1%, 2%, 3%, and 4% for treating cells for 8, 16, 24, and 32 h. In the RNase R assay, the total RNA of the same mass was prepared and divided into two groups. For the RNase R treatment group, the total RNA was incubated with 2 U/μg RNase R (Beyotime Biotechnology, R7092S) at 37 °C for 15 min. Total RNA was incubated with RNase R Reaction Buffer as the control group. In the actinomycin D assay, 2 μg/mL actinomycin D (MedChemExpress, HY-17559) was added to the cells and cultured at 37 °C in an environment with 5% carbon dioxide for specific times. For CHX treatment, cells were incubated with 40 µg/mL CHX (ABMole, M4879) at the indicated time points.

### Quantitative real-time PCR (RT-qPCR)

Plasmids or siRNAs were transfected according to the experimental design. Transfected cells were mixed with total RNA extraction reagent (Epizyme, YY101) and then added with chloroform for total RNA extraction. The RNA samples of equal mass were synthesized to obtain the cDNA samples by using the RT Mix Kit (AG Biotechnology, AG11728). The Hieff qPCR SYBR Green Master Mix Kit was used to perform RT-qPCR experiments. Table S1 (Supporting Information) presents all the primer information (Sangon Biotech, China).

### Cell Counting Kit-8 (CCK-8) assay

Treated cells were diluted to a density of 1000 cells per 100 μL medium and cultured in 96-well plates for specific times. CCK-8 solution (CellorLab, CX001S) was added to wells and incubated at 37 °C for 1 h. Cell viability was evaluated at an absorbance of 450 nm by a multifunctional microplate reader (PerkinElmer, 1701227S).

### Fluorescence in situ hybridization (FISH) and immunofluorescence staining

The treated cells were cultured in a confocal dish (NEST, 801002). 0.2% Triton X-100 (Biosharp, BL2202A) permeabilized cells for 12 min after fixing cells with 4% paraformaldehyde (Biosharp, BL539A). For the FISH assay, cells were incubated with a FAM-labeled circPDE4D probe (GenePharma, 25237448) at 37 °C for 12 h. In immunofluorescence experiments, corresponding primary antibodies were added to cells and incubated at 4 °C for 12 h. After that, cells were incubated with the secondary antibodies at 37 °C for 1 h. DAPI and anti-fade mounting medium were added to the cells. The images were captured by a laser confocal microscope (LSM980, Carl Zeiss, Oberkochen, Germany).

### Flow cytometry assay

The treated cells were resuspended with binding buffer and collected in a flow cytometry tube, then the cells were stained using the Annexin V-FITC/PI Apoptosis Kit (APExBIO, K2003). The signals were detected by a flow cytometer (Beckman, CytoFlex).

### Western blot

Transfected cells were lysed in RIPA lysis buffer (Beyotime Biotechnology, P0013B), which was supplemented with protease inhibitor (Beyotime Biotechnology, ST505) for protein extraction. Identical protein concentrations were loaded into an SDS-PAGE gel for protein electrophoresis and then transferred onto PVDF membranes (Millipore, ISEQ00005). The membranes were incubated with the indicated primary antibodies with rotation at 4 °C overnight after sealing with 5% skim milk (Biosharp, BS102). The corresponding secondary antibodies were added to these membranes for 1 h. Finally, protein signals were detected by a luminescent imaging system (Tanon, 5200). All antibodies used in this study are listed in Table S2 (Supporting Information).

### ELISA assay

The samples of cell supernatant and BALF of mice, as well as the ELISA Kits, were placed at room temperature for balancing before detection. The Human ELISA Kits were used to evaluate the IL-1β (Multi Sciences, EK101B) and IL-6 (Proteintech, KE00385) levels of supernatant from the treated cells. The Mouse ELISA Kits were used to evaluate the IL-1β (Multi Sciences, EK201B) and IL-6 (Multi Sciences, P08505) levels of the BALF from mice according to the manufacturer’s instructions. Briefly, the samples were added to the ELISA plate, and then the antibody was incubated for 2 h. After that, the enzyme conjugate working solution was added to the ELISA plate for incubation. OD values were evaluated at absorbance of 450 and 630 nm by a multifunctional microplate reader (PerkinElmer, 1701227S).

### Dual luciferase reporter assay

Dual-luciferase reporter plasmids (WT and MUT) of circPDE4D and SMURF1 were synthesized by GenePharma. Transfected cells were cultured in 12-well plates for 48 h and then lysed using the Dual Luciferase Reporter Gene Assay Kit II (Beyotime Biotechnology, RG029S). Firefly and Renilla luciferase levels were detected using the multifunctional microplate reader (PerkinElmer, 1701227S).

### Co-Immunoprecipitation (Co-IP)

Treated cells were lysed using RIPA buffer (Beyotime Biotechnology, P0013B), which contained protease inhibitor. The control IgG and the anti-SMURF1 were mixed with cell lysates and then incubated under constant rotation at 4 °C overnight. Protein A + G beads (Thermo Fisher Scientific, 88802) were used to absorb the immunoprecipitate. Western blot analysis was performed to detect the interaction of proteins.

### Fast silver staining

According to the manufacturer’s instructions of the Fast Silver Stain Kit (Beyotime Biotechnology, P0017S), the SDS-PAGE gels were washed with 30% ethanol after fixing overnight. The gels were stained with silver solution for 10 min, and then were colored using the chromogenic solution. Finally, the gels were collected for Mass spectrometry (MS) assays.

### Mass spectrometry (MS) analysis

The peptide samples obtained from HBEC cells were detected and analyzed on Orbitrap Eclipse by QLBio Co., Ltd. Briefly, the gel was cut into pieces, which were then dehydrated by using acetonitrile and treated with dithiothreitol. These gel pieces were used to obtain the peptides through the enzymatic digestion after alkylation with iodoacetamide. The results were analyzed and collected by using MaxQuant software version v1.6.2.10.

### Adeno-associated virus (AAV) construction

FITC-conjugated AAV-circPDE4D and FITC-conjugated AAV-Vector were generated by Genomeditech (Shanghai, China). The target plasmids were obtained from the rAAV expression plasmid, which contained the sequence of circPDE4D. Then, the circPDE4D plasmids were transfected to construct the circPDE4D-AAV pro-293T cells. The circPDE4D-AAV pro-293T cells were rich in high-titer viruses, and concentrated and purified to generate AAV virus (5.02 × 10^+12^ VG/mL). In animal models, the mice which exposure to CS for 5 months were intratracheally instilled with the AAV virus (3 × 10^+11^ VG per mouse). Finally, the lung tissues of mice were cut into lung sections, and the green fluorescence of the lung sections was observed to evaluate the effect of virus infection.

### Animal experiments

C57BL/6 male mice of 8 weeks were randomly divided into four groups: control (Con), CS, AAV-Vector+CS, AAV-circPDE4D+CS (*n* = 6 mice per group). The control group was normally exposed to air. For CS groups, mice were placed in a purpose-built whole-body inhalation chamber containing uniformly dispersed tobacco particles under the following conditions: twice a day, 90 min each time, with a 4-h smoke-free recovery period between exposures and 6 consecutive exposure days followed by 1 day of recovery, for 6 months. For AAV-circPDE4D+CS and its control group, the mice which exposure to CS for 5 months were intratracheally instilled with FITC-labeled AAV-circPDE4D and FITC-labeled AAV-Vector virus (3 × 10^+11^ VG per mouse). All mice were bred with regular water and food in the Animal Experiment Center of Anhui Medical University. After 6 months, an appropriate amount of pentobarbital sodium (50 mg/kg) was used to euthanize all mice. Then, the lung tissues of mice were obtained for further experiments and blind analysis.

### Histological staining and immunohistochemistry (IHC)

Lung tissue sections of mice were baked in a constant temperature box and then dewaxed with xylene. The lung sections were rehydrated using graded ethanol. The H&E staining solution (Biosharp, BL700A) was used to perform the H&E experiments, PAS staining was conducted using a commercial kit (Biosharp, BL1120B), and Masson reactions were developed according to the manufacturer’s protocol (Biosharp, BL1059A). For IHC staining, the lung tissue sections were incubated with 3% hydrogen peroxide to inactivate the endogenous peroxidase activity and then soaked in sodium citrate solution. After being treated with goat serum blocking solution, the sections were incubated with anti-SMURF1 at 4 °C overnight, and then incubated with the secondary antibody. Microscopic imaging was performed using the VS200 system (Olympus, Tokyo, Japan).

### Bioinformatics analysis

The circBase database (http://www.circbase.org/) was utilized to analyze circPDE4D sequences. The target miRNAs of circPDE4D were predicted by circinteractome (https://circinteractome.nia.nih.gov) and circBase. Target genes were predicted by TargetScan (https://www.targetscan.org/).

### Statistical analysis

Experiments were repeated with at least three biological replicates. Statistical analysis was performed by GraphPad Prism 8.0. For differences between the two groups, two independent sample t-tests were used to assess statistical significance. For multi-group comparisons, one-way or multi-factor analysis of variance (ANOVA) was used. Correlation analysis was conducted using Pearson’s correlation test. Data are presented as mean ± SD values. Statistical significance was defined as a *P*-value less than 0.05.

## Supplementary information

Original data of Western blot

Supplemental Material of Figures and Tables

## Data Availability

Data available on request from the authors. The uncropped western blotting imprints are uploaded as Supplementary Material.
