# Supplementary material for: K48 and K63 linkage-competed ubiquitination of BECN1 promotes circPDE4D-mediated autophagy in chronic obstructive pulmonary disease
Source: Cell Death Dis. 2026 Mar 19;17(1):321. doi: 10.1038/s41419-026-08582-8 (PMC13039329; doi:10.1038/s41419-026-08582-8)

**Original data of Western Blot**

RAW_Fig. 2.


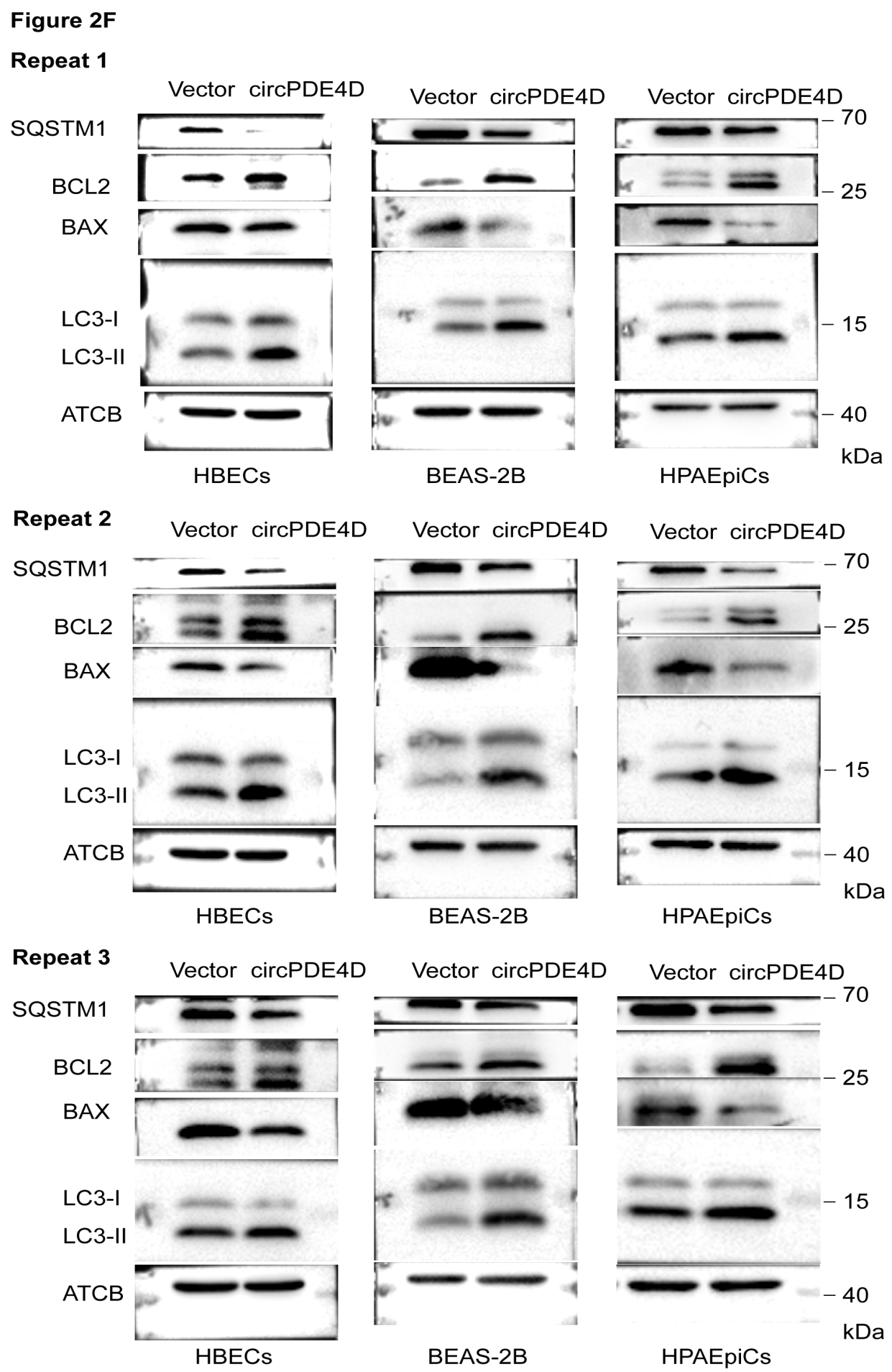


RAW_Fig. 4 and 5M.


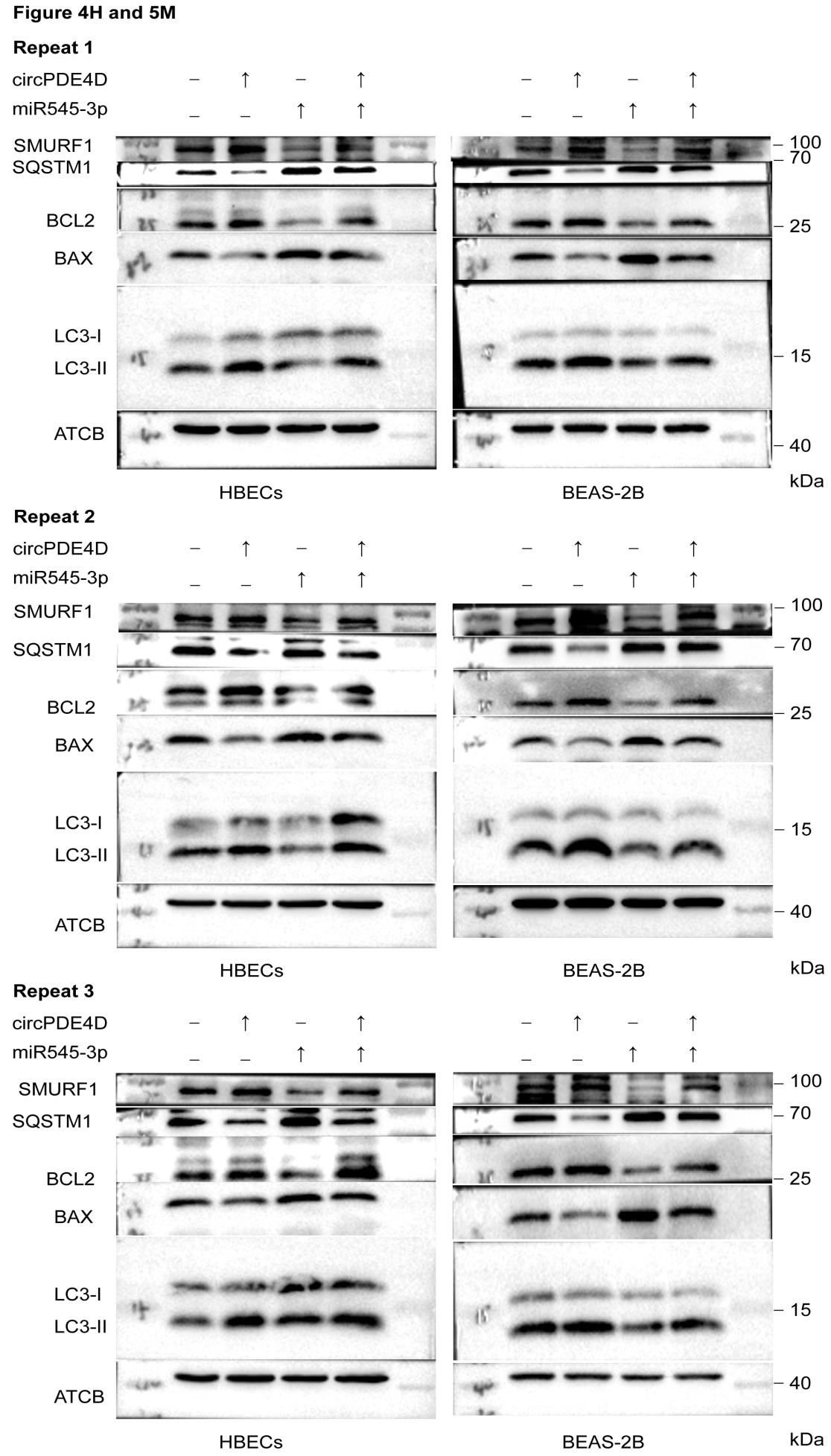


RAW_Fig. 5.


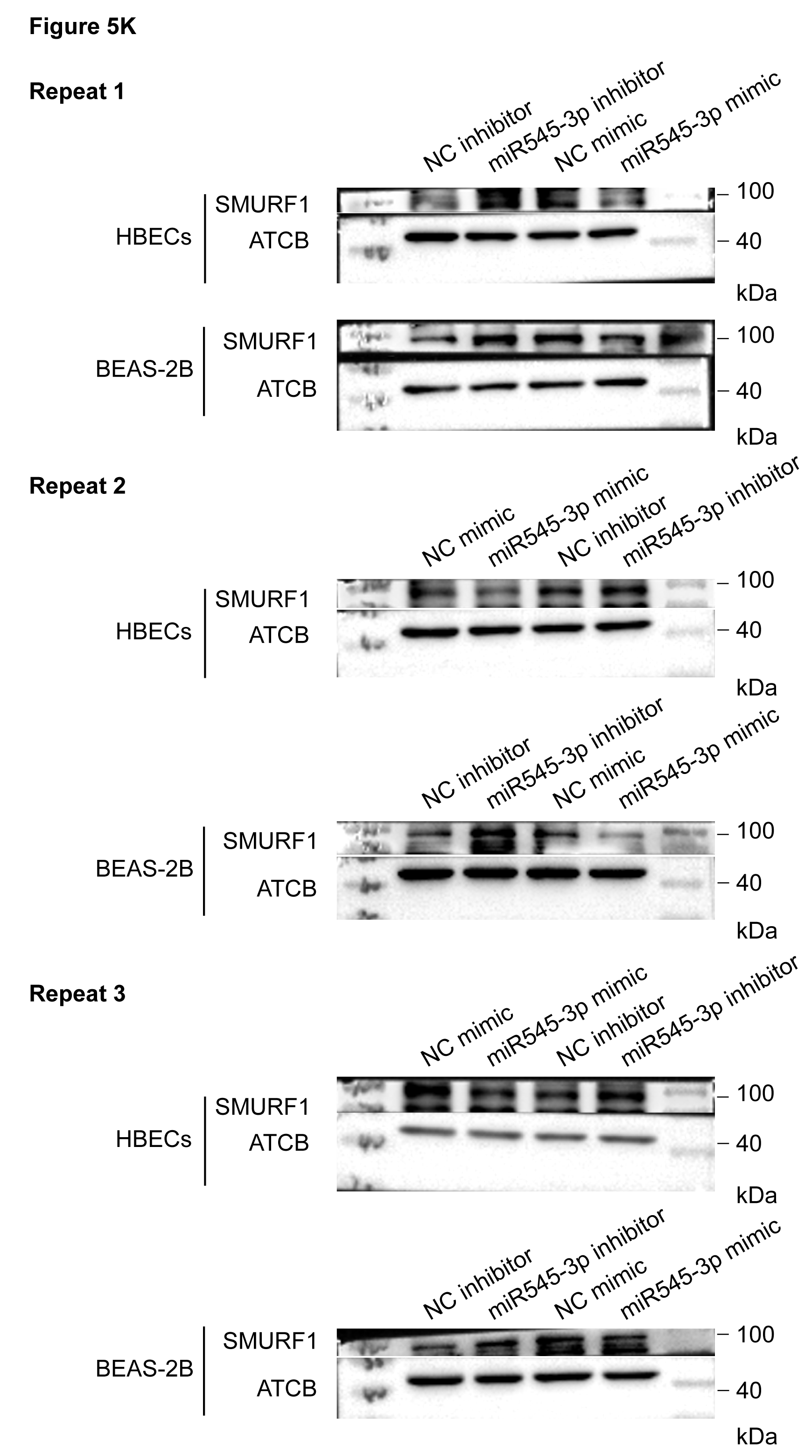


RAW_Fig. 5M and S5G.


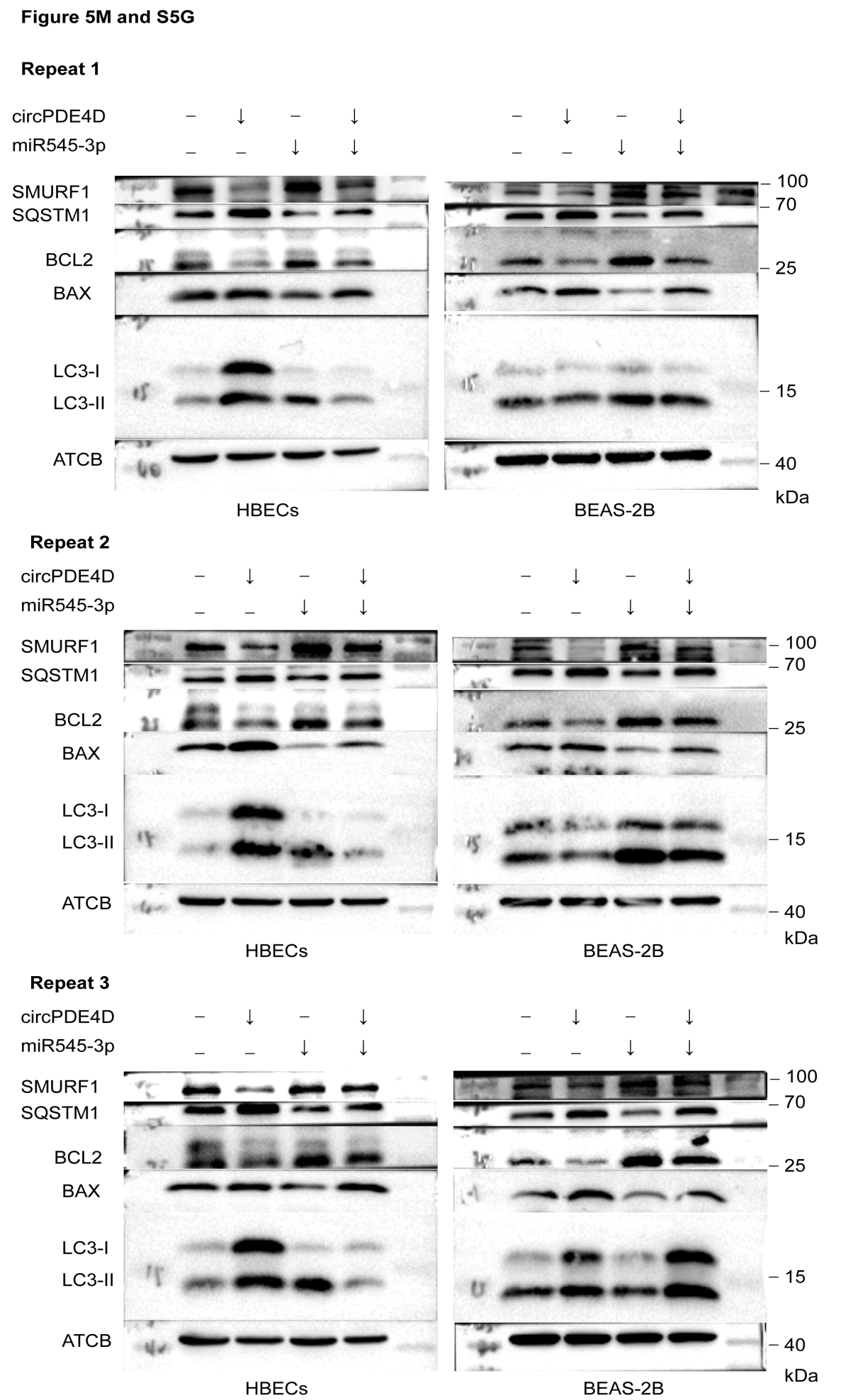


RAW_Fig. 6.


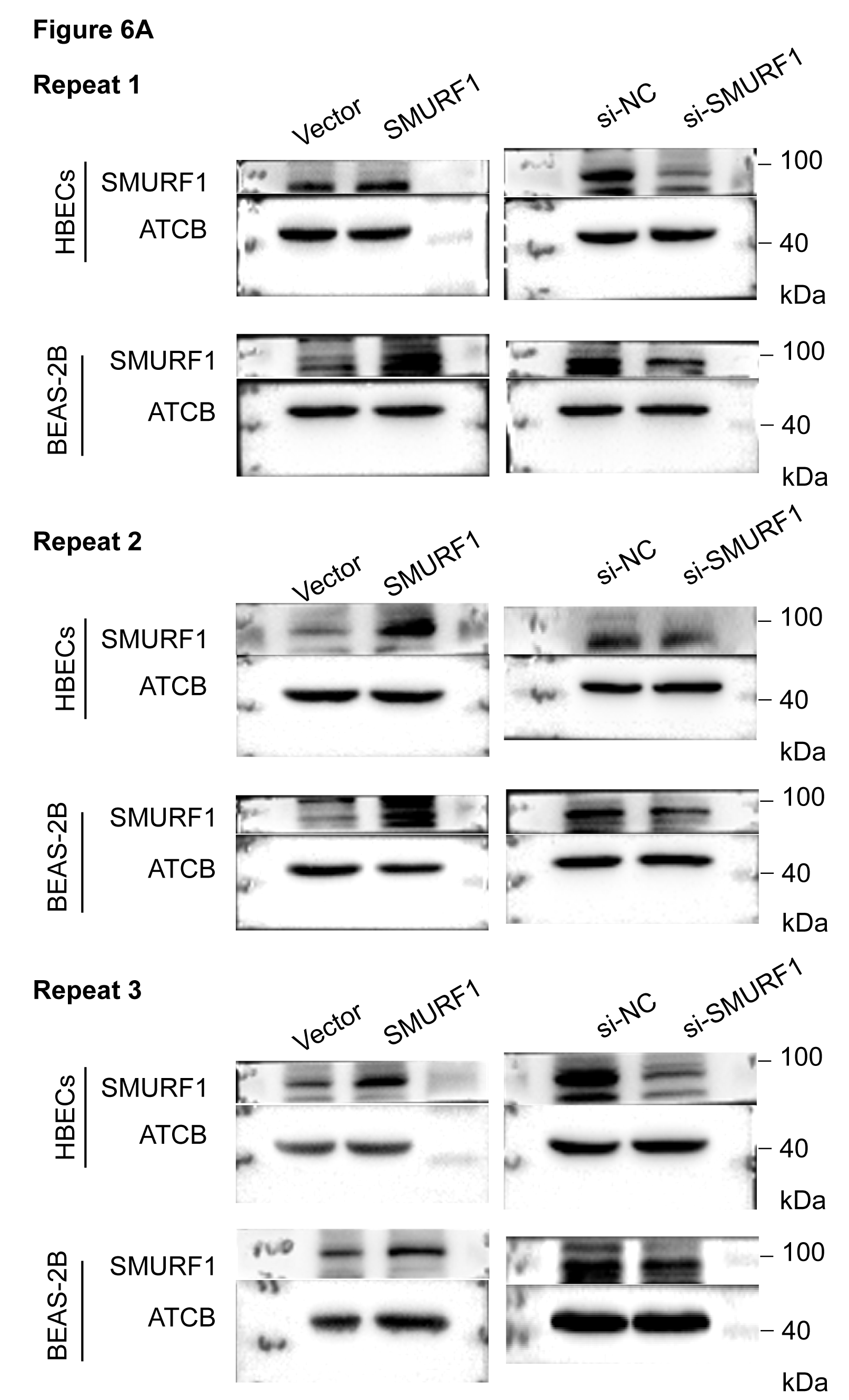


RAW_Fig. 6.


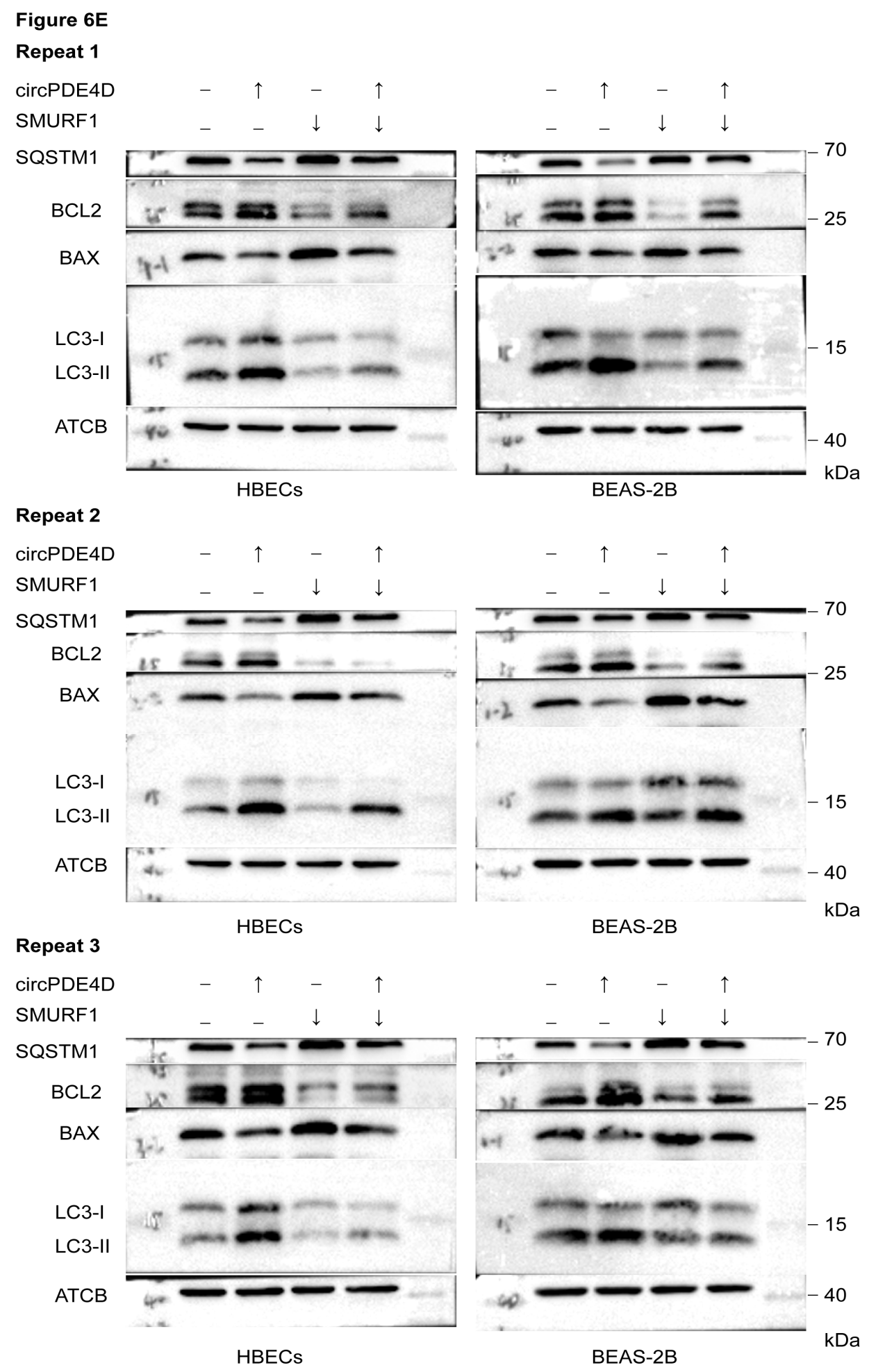


RAW_Fig. 7.


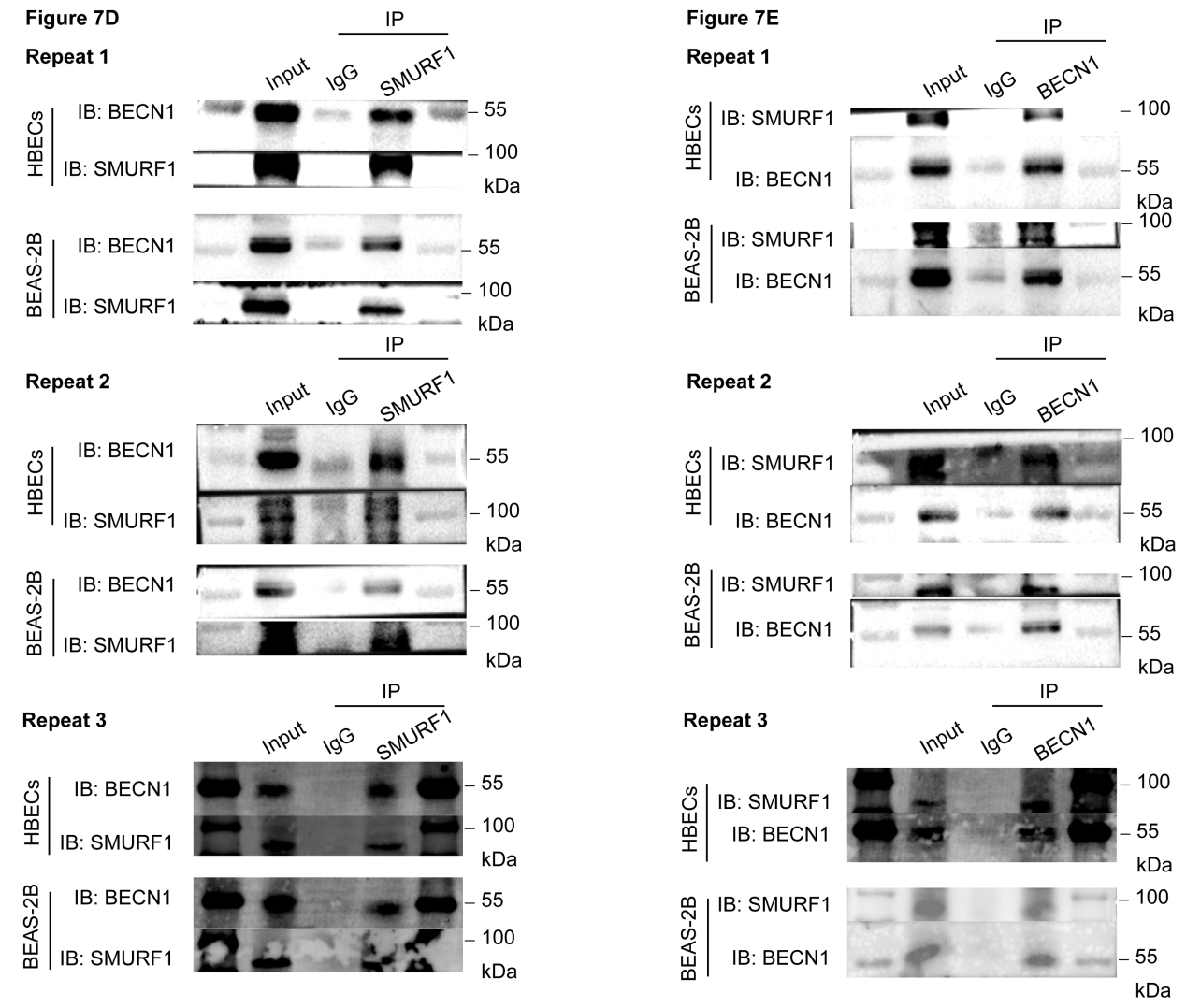


RAW_Fig. 7.


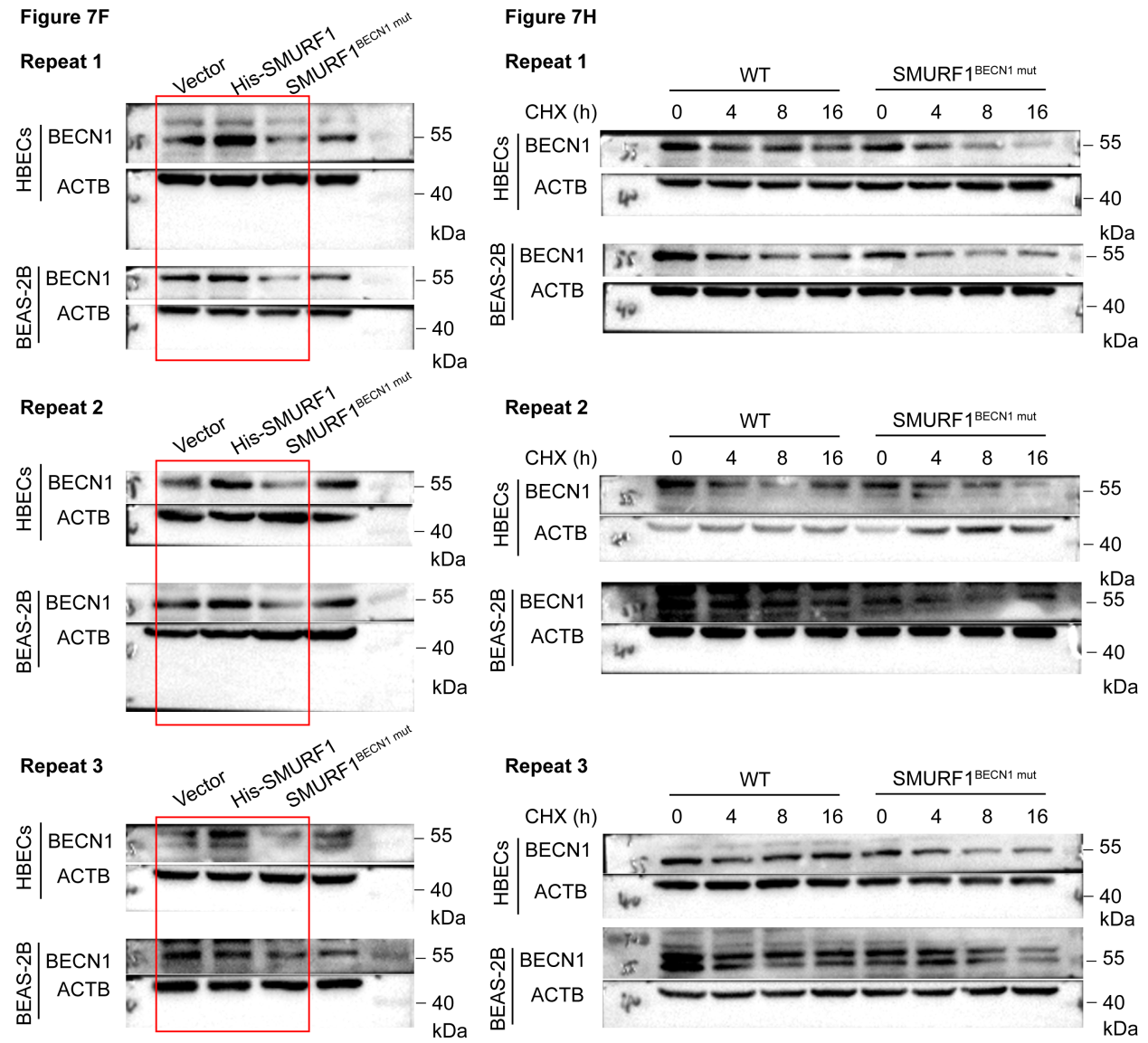


RAW_Fig. 7.


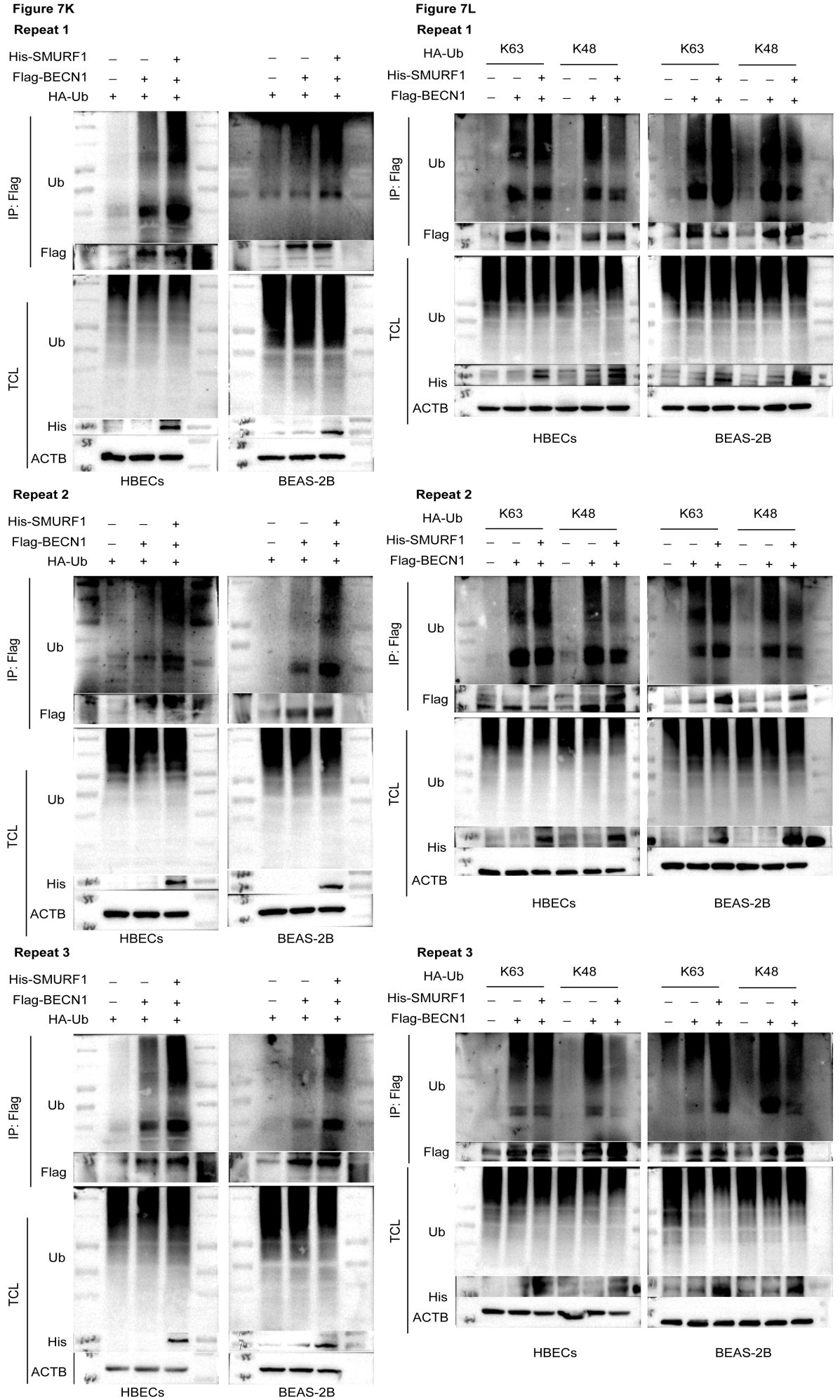


RAW_Fig. 8.


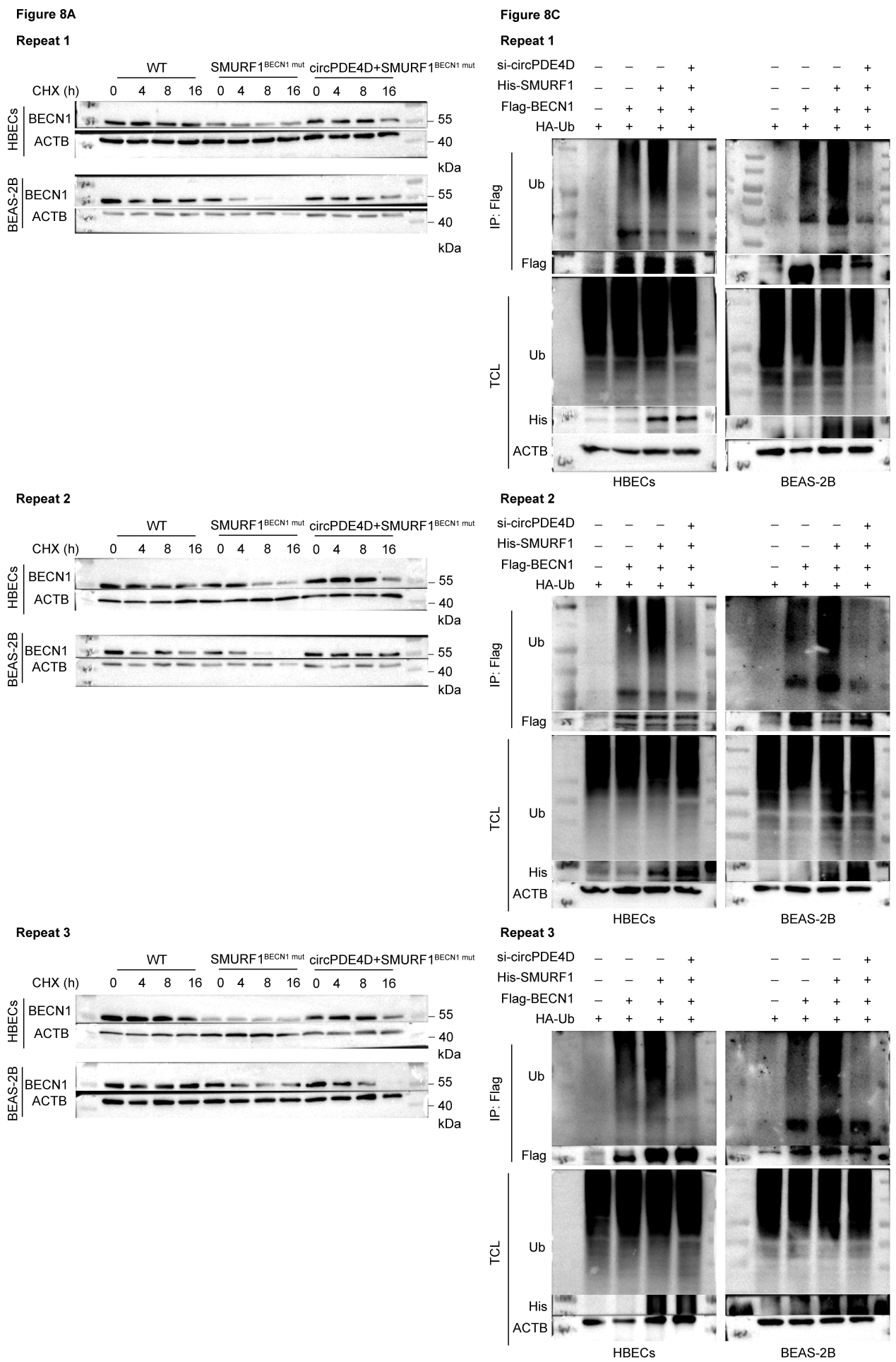


RAW_Fig. 8.


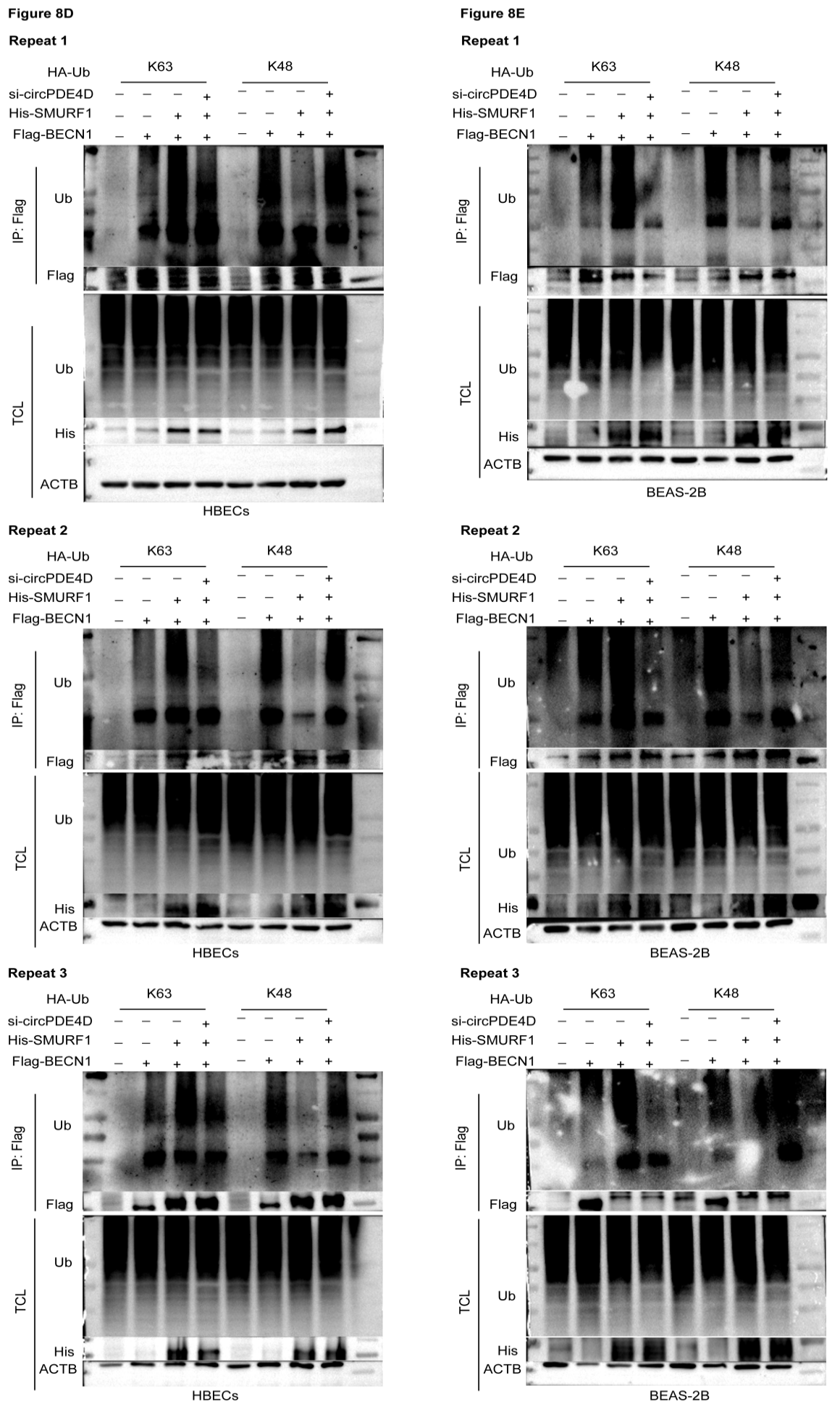


RAW_Fig. S2.


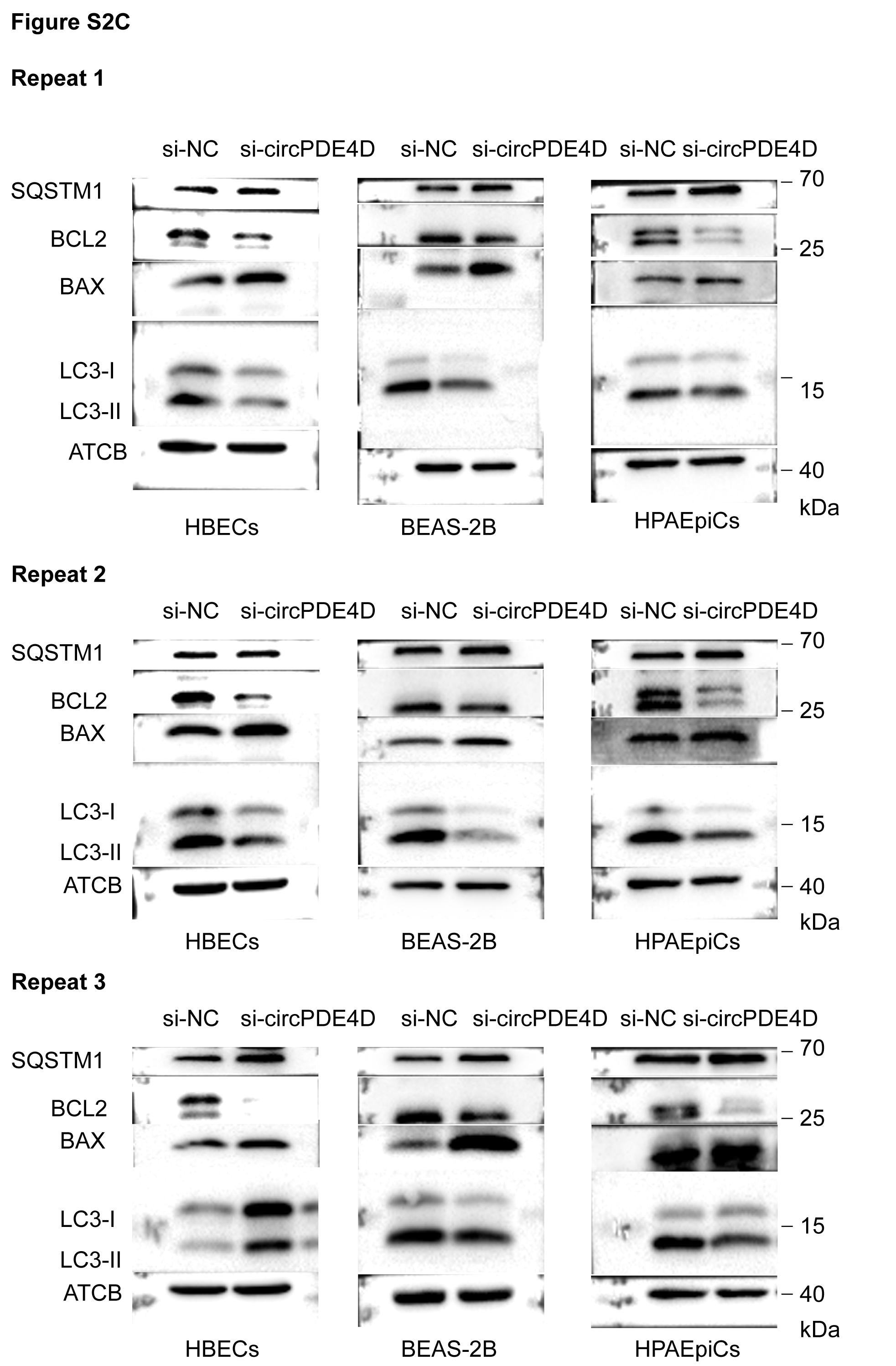


RAW_Fig. S7.


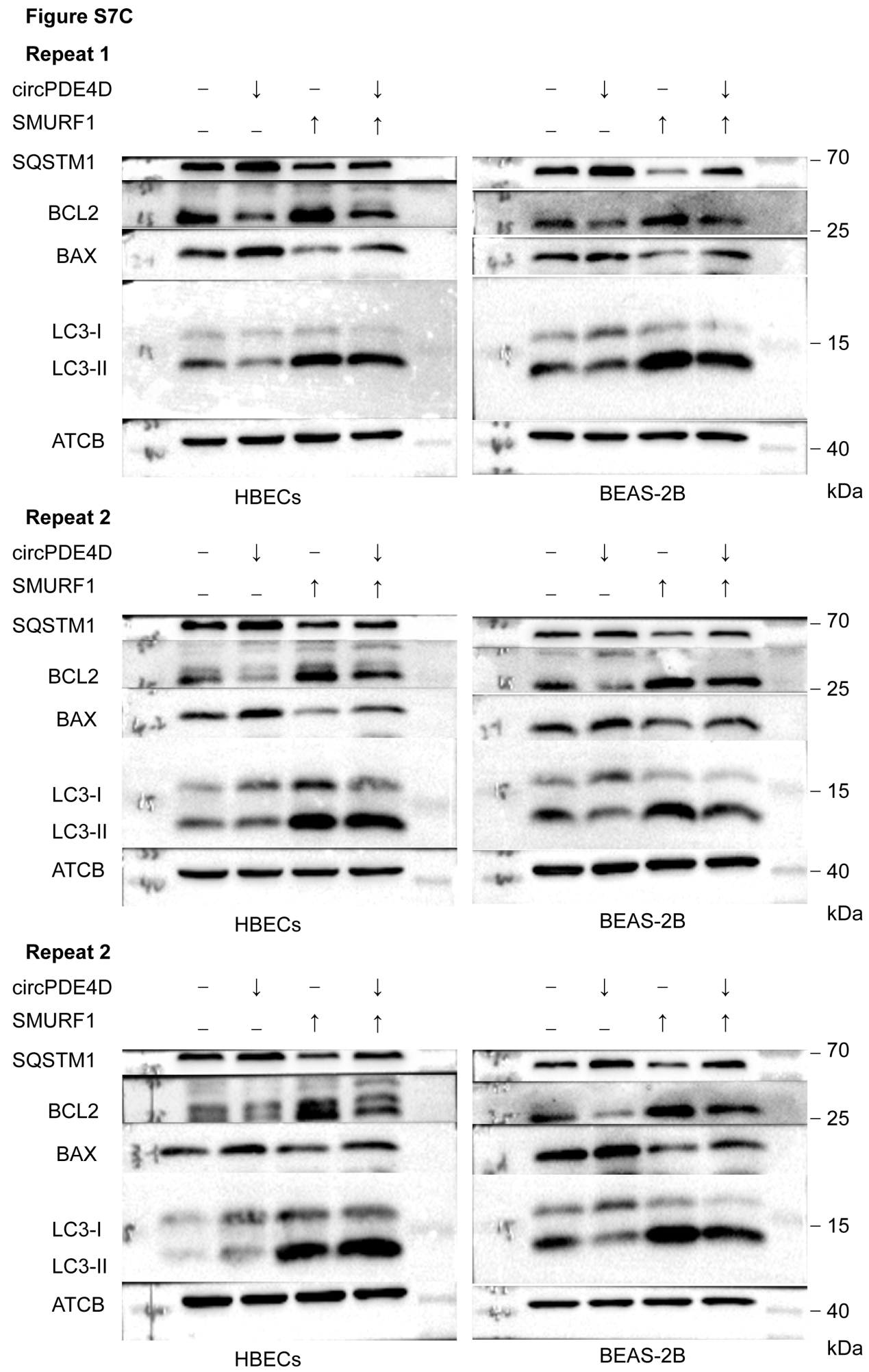

Supplement: Supplementary file 1 — Original data of Western blot [file 41419_2026_8582_MOESM1_ESM.docx]
