# Supplementary material for: K48 and K63 linkage-competed ubiquitination of BECN1 promotes circPDE4D-mediated autophagy in chronic obstructive pulmonary disease
Source: Cell Death Dis. 2026 Mar 19;17(1):321. doi: 10.1038/s41419-026-08582-8 (PMC13039329; doi:10.1038/s41419-026-08582-8)
Supplement: Supplementary file 2 — Supplemental Material of Figures and Tables [file 41419_2026_8582_MOESM2_ESM.pdf]

Supplemental Material of Figures:

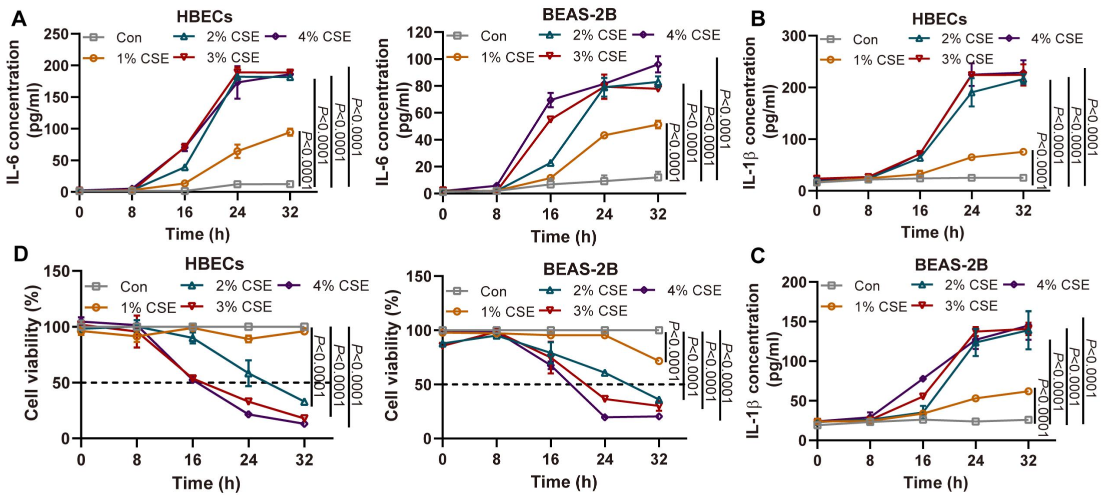

Fig. S1. CSE-induced cells are generated. (A-C) ELISAs of IL-6 (A) and IL-1β (B-C) levels in HBECs and BEAS-2B cells after CSE treatment at several concentrations and for different times. (D) CCK-8 assays of cell viability in HBECs and BEAS-2B cells with the indicated treatment. Data are presented as mean ± SD values.

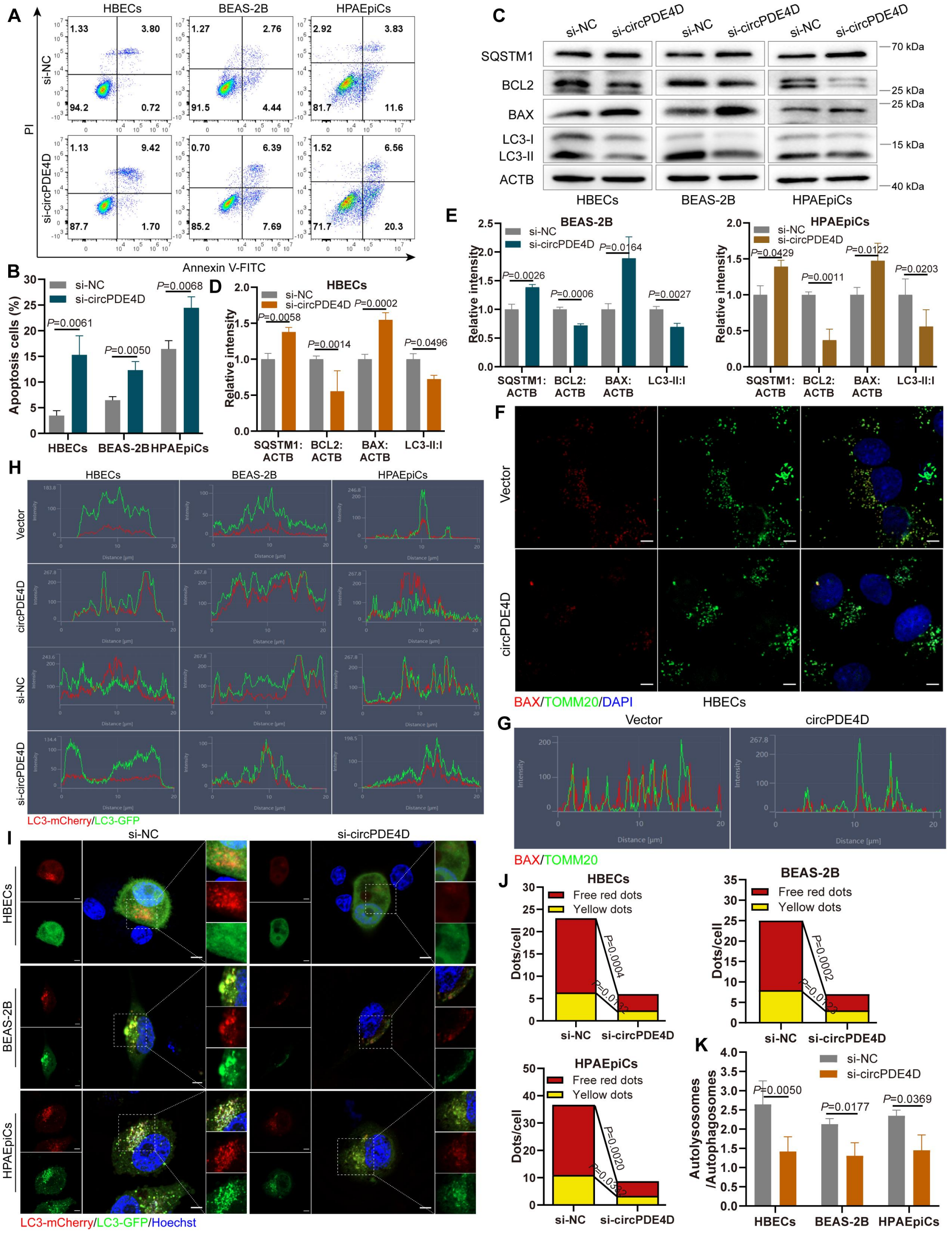

Fig. S2. CircPDE4D knockdown promotes apoptosis and suppresses autophagy *in vitro*. (A-B) Flow cytometry assays and statistical quantification of apoptosis in cells after silencing circPDE4D. (C-E) Western blotting with quantitative analysis of SQSTM1/p62, BCL2, BAX, and LC3 in cells after circPDE4D knockdown. (F-G) Immunofluorescence images of BAX and TOMM20 in circPDE4D overexpression

and control groups in HBECs cells. Nuclei were stained with DAPI. 5 µm scale bar. (H-K) LC3 dots as reflected by free red dots and yellow dots, and quantitative analyses in transfected mCherry-GFP-LC3 adenovirus cells with indicated treatments. Nuclei were stained with Hoechst. 5 µm scale bar. Data are shown as means ± SD.

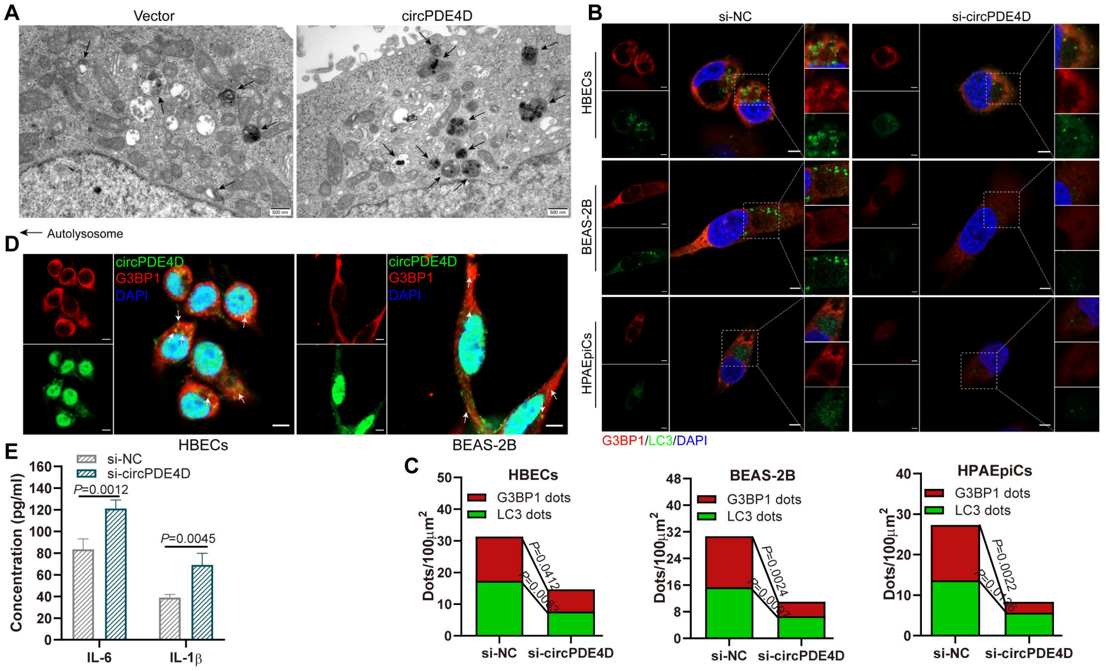

Fig. S3. CircPDE4D regulates autophagy and inflammation *in vitro*. (A) Autolysosomes were detected by transmission electron microscopy in HBECs cells with circPDE4D overexpression and its control treatments. 500 nm scale bar. (B-C) G3BP1 dots (red) and LC3 dots (green) following experimental treatments were detected by Immunofluorescence staining. Nuclei were stained with DAPI. 5 µm scale bar. (D) Co-localization of circPDE4D FISH (green) and G3BP1 (red) in cells. Nuclei were stained with DAPI. 5 µm scale bar. (E) ELISA detection of the concentrations of IL-6 (in BEAS-2B) and IL-1β (in HBECs) in si-circPDE4D and its control groups. Data are shown as means ± SD.

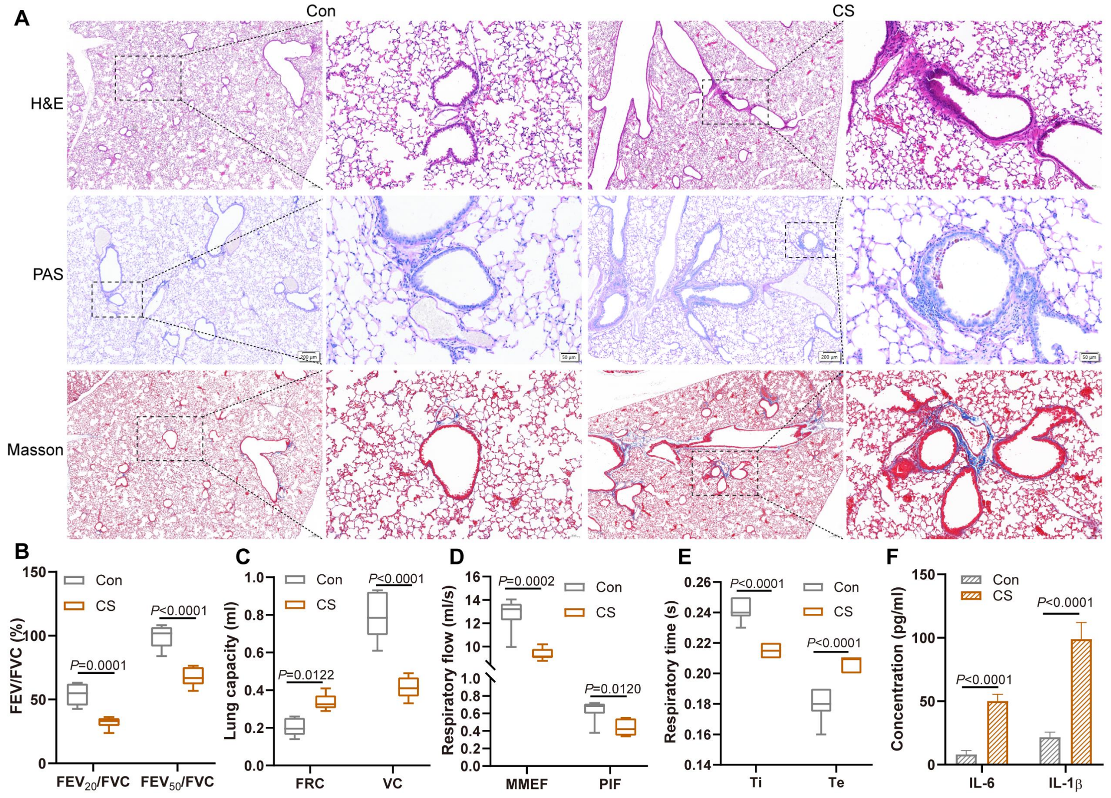

Fig. S4. The construction of a mouse model of CS exposure. (A) Representative images of H&E, PAS, and Masson staining of mouse lungs in control and CS groups (n = 6 mice per group). (B-E) The indicated lung function indices of the two groups. (F) IL-6 and IL-1β levels were detected by ELISA assay in the control and CS groups. Data are presented as mean ± SD values.

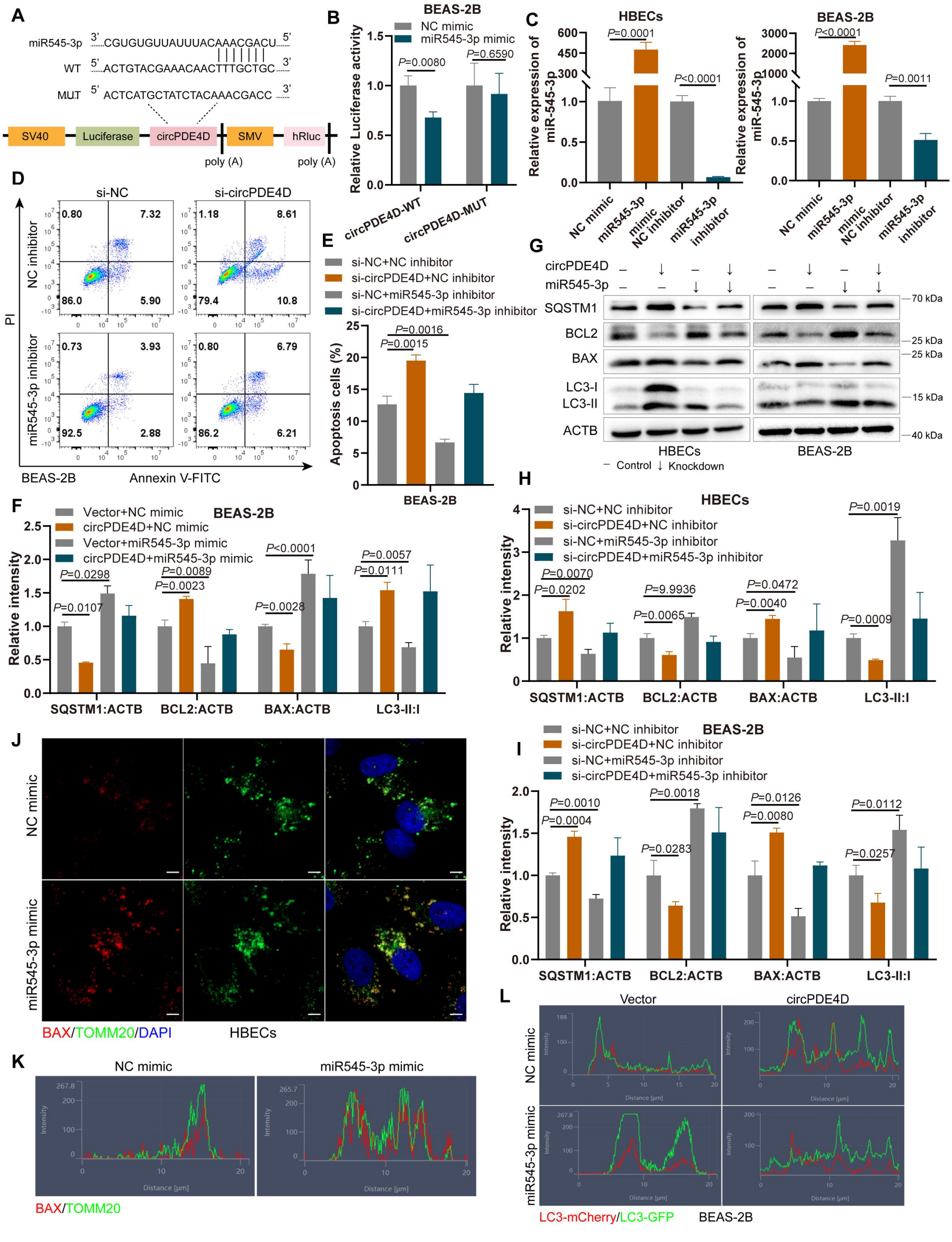

Fig. S5. CircPDE4D targets miR545-3p to regulate cell apoptosis. (A) Schematic of the luciferase reporter plasmids of circPDE4D-WT and MUT. (B) Relative luciferase activities in BEAS-2B cells in the indicated groups. (C) Relative expression of miR545-3p in cells transfected with miR545-3p mimic and inhibitor or their controls. (D-E) Apoptosis in BEAS-2B cells transfected as indicated was detected by

flow cytometry assay. (F-I) Western blot analysis and quantitation of SQSTM1/p62, BCL2, BAX, and LC3 proteins in BEAS-2B cells with indicated treatments. (J-K) Immunofluorescence experiments of BAX and TOMM20 in HBECs cells with miR545-3p mimic and its control treatments. 5 μm scale bar. (L) Quantitation of LC3-mCherry and LC3-GFP in the indicated groups. Data are shown as means ± SD.

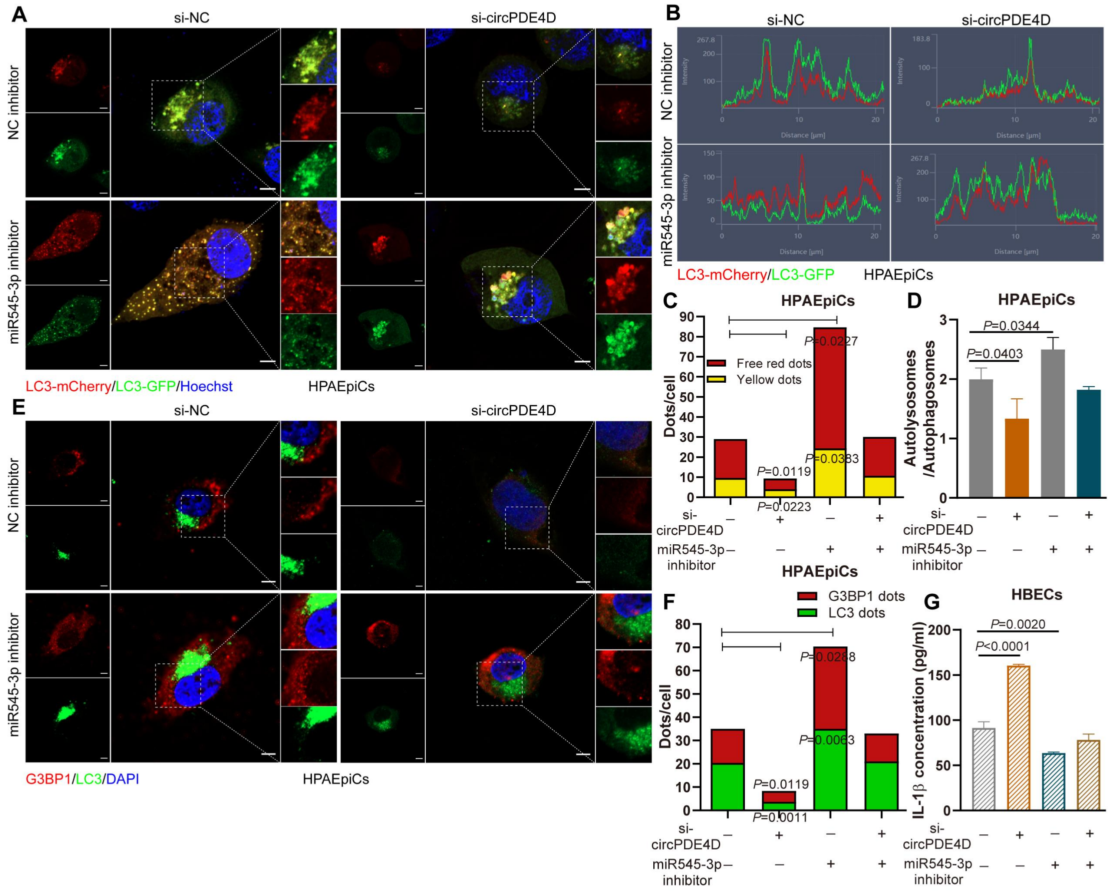

Fig. S6. CircPDE4D-miR545-3p axis regulates cell autophagy, SG formation, and the release of IL-1β. (A-D) LC3 dots reflected by free red dots and yellow dots were evaluated after mCherry-GFP-LC3 transfection in HPAEpiCs cells with indicated treatments. Nuclei were stained with Hoechst. 5 μm scale bar. (E-F) Immunofluorescence experiments of G3BP1 dots (red) and LC3 dots (green) in HPAEpiCs cells in several groups. Nuclei were stained with DAPI. 5 μm scale bar. (G) ELISA detection of IL-1β levels in HBECs cells in the indicated groups. Data are shown as means ± SD.

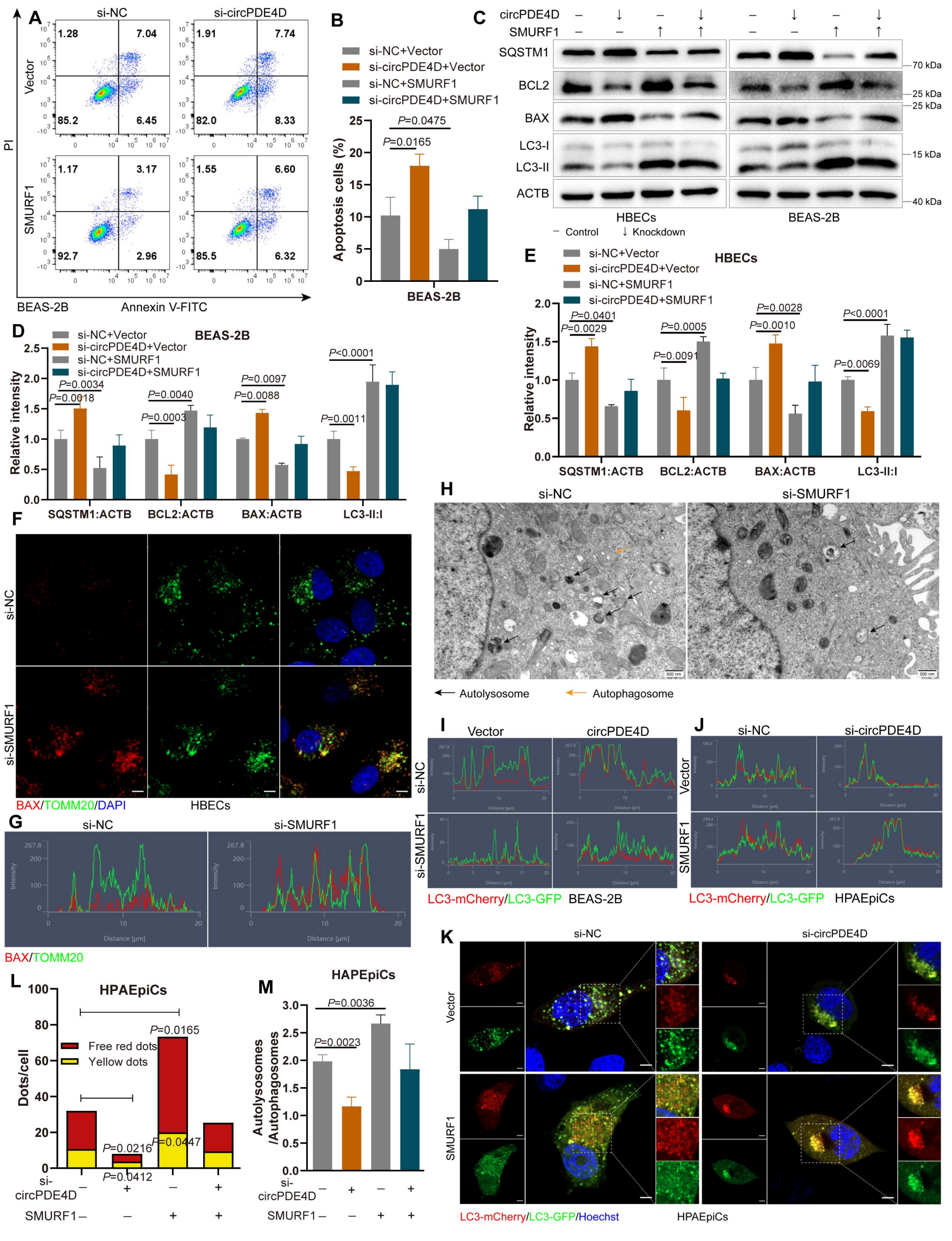

Fig. S7. The effects of circPDE4D-SMURF1 axis on apoptosis and autophagy. (A-B) Apoptosis in BEAS-2B cells with indicated treatments was detected by flow cytometry assay. (C-E) Western blot analysis and quantitation of SQSTM1/p62, BCL2, BAX, and LC3 proteins in HBECs and BEAS-2B cells in the indicated groups. (F-G) Co-localization and quantification of BAX and TOMM20 in HBECs cells with

si-SMURF1 and its control transfection. (H) Autolysosomes and autophagosome were detected by transmission electron microscopy in HBECs cells after SMURF1 knockdown. 500 nm scale bar. (I-M) LC3 dots and quantitative analyses in HPAEpiCs cells in the indicated groups. Hoechst dye was used to stain cell nuclei. Scale bar: 5 μm. Data are presented as mean ± SD values.

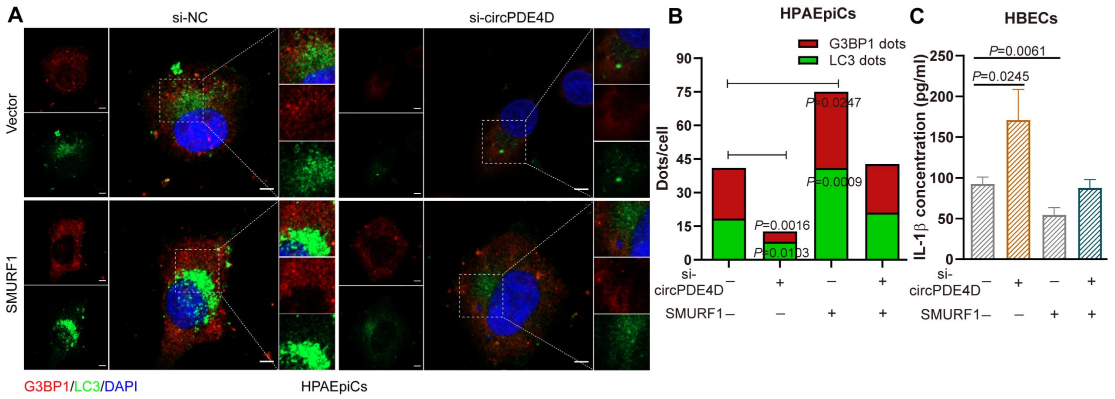

Fig. S8. The effects of circPDE4D-SMURF1 axis on SG formation and IL-1β release. (A-B) G3BP1 dots (red) and LC3 dots (green) in HPAEpiCs cells were evaluated by immunofluorescence assay. DAPI was used to stain cell nuclei. Scale bar: 5 μm. (C) IL-1β levels were detected by ELISA assays in HBECs cells in the indicated groups. Data are presented as mean ± SD values.

Supplemental Material of Tables:

Table S1. Primers, oligo, probes, and plasmids used in this study.	
**Gene**	**Sequence (5'-3') or supplier**
β-actin	F: AGCGAGCATCCCCCAAAGTT
	R: GGGCACGAAGGCTCATCATT
U6	F: CTCGCTTCGGCAGCACA
circPDE4D	F: AGCCACCATAACAGTTTTGATGT
	R: GGAGTTCCGGGACATAGACT
linear *PDE4D*	F: AACTCCTCCATTGCCAGTGA
	R: TGGATGGTTGGTTGCACATG
miR545-3p	UCAGCAAACAUUUAUUGUGUGC
miR382	GCUUAGGUGGUGCUUGUUGAAG
miR769-3p	UUGGUUCUGGGGCCUCUAGGGUC
SMURF1	F: TCTTCAATCAACCCCGACCA
	R: AAGGTGTGGTCCAGTACAGG
Negative control	sense: UUCUCCGAACGUGUCACGUTT
	antisense: ACGUGACACGUUCGGAGAATT
si-circPDE4D	sense: AGCCACCAUAACAGUUUUGTT
	antisense: CAAAACUGUUAUGGUGGCUTT
miR545-3p mimic	sense: UCAGCAAACAUUUAUUGUGUGC
	antisense: ACACAAUAAAUGUUUGCUGAUU
NC inhibitor	sense: CAGUACUUUUGUGUAGUACAA
miR545-3p inhibitor	sense: GCACACAAUAAAUGUUUGCUGA
si-SMURF1	sense: CCAGCACUAUGAUCUAUAUTT
	antisense: AUAUAGAUCAUAGUGCUGGTT
circPDE4D fish probe with FAM conjugated	CCATTGTCCACATCAAAACTGTTATGGTGGCTTTGTTG
Plasmid: *circPDE4D*	Gene Pharma
Plasmid: *SMURF1* pcDNA 3.1	Gene Pharma
Plasmid: His-*SMURF1*	TsingKe
Plasmid: Flag-*BECN1*	TsingKe
Plasmid: His-$SMURF1^{BECN1mut}$	TsingKe
Plasmid: HA-*Ub*	BioGo
Plasmid: HA-*Ub-K48*	BioGo
Plasmid: HA-*Ub-K63*	BioGo

**Table S2.** *Antibodies used in the present study.*		
**Product**	**Source**	**No. of Catalogue**
**Western blot:**		
anti-β-actin	HUABIO	EM21002
anti-SMURF1	Boster	PB0937
anti-BECN1	Abmart	T55092
anti-G3BP1	Proteintech	66486-1-Ig
anti-LC3	Cell Signaling Technology	12741S
anti-BCL2	Cell Signaling Technology	4223S
anti-BAX	Cell Signaling Technology	36030S
anti-SQSTM1	Abmart	T55546
anti-HA-Tag	Abmart	R20003
anti-DYKDDDK Tag	Abmart	R20008
anti-His-Tag	Proteintech	66005-1-Ig
anti-Ubiquitin	Diagbio	db15832
anti-TOMM20	Immunoway	YT4696
**Secondary antibody**		
Anti-rabbit/mouse IgG, HRP-linked Antibody	Abmart	M21003S
Anti-rabbit IgG, HRP-linked Antibody	Cell Signaling Technology	7074P2
Anti-mouse IgG, HRP-linked Antibody	Cell Signaling Technology	7076P2
Goat Anti-Rabbit lgG AF 594	Abmart	M21014
Goat Anti-Mouse lgG AF 594	Abmart	M21013
Goat Anti-Mouse lgG AF 488	Abmart	M21011
